# Three-dimensional nanoimaging of fuel cell catalyst layers

**DOI:** 10.1038/s41929-023-00947-y

**Published:** 2023-04-17

**Authors:** Robin Girod, Timon Lazaridis, Hubert A. Gasteiger, Vasiliki Tileli

**Affiliations:** 1 Institute of Materials, École Polytechnique Fédérale de Lausanne, Lausanne, Switzerland; 2 Chair of Technical Electrochemistry, Department of Chemistry and Catalysis Research Center, Technische Universität München, Garching, Germany

**Keywords:** Energy, Electrocatalysis, Fuel cells, Imaging studies

## Abstract

Catalyst layers in proton exchange membrane fuel cells consist of platinum-group-metal nanocatalysts supported on carbon aggregates, forming a porous structure through which an ionomer network percolates. The local structural character of these heterogeneous assemblies is directly linked to the mass-transport resistances and subsequent cell performance losses; its three-dimensional visualization is therefore of interest. Herein we implement deep-learning-aided cryogenic transmission electron tomography for image restoration, and we quantitatively investigate the full morphology of various catalyst layers at the local-reaction-site scale. The analysis enables computation of metrics such as the ionomer morphology, coverage and homogeneity, location of platinum on the carbon supports, and platinum accessibility to the ionomer network, with the results directly compared and validated with experimental measurements. We expect that our findings and methodology for evaluating catalyst layer architectures will contribute towards linking the morphology to transport properties and overall fuel cell performance.

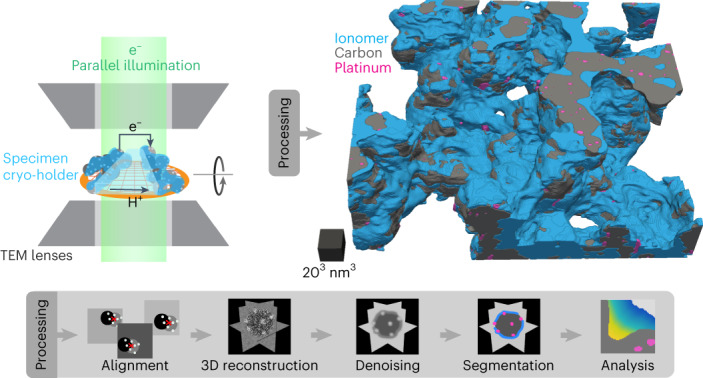

## Abstract (web summary)

The catalyst layer in proton-exchange membrane fuel cells involves the complex and crucial interplay between an ionomer network and metallic nanoparticles supported on carbons, but current methods are unable to describe it with high resolution. Now electron tomography at cryogenic temperatures and deep learning algorithms are used to provide quantitative three-dimensional imaging at nanometre resolution of a fuel cell catalyst layer structure.

## Main

The design of catalyst layers for proton-exchange membrane fuel cells involves an optimal interplay between metallic nanoparticles (2–3 nm, heavy-element and crystalline) and the carbon supports (mostly amorphous, porous structures) in relation to the coverage provided by an ionomer network (down to a couple of nanometres, amorphous and highly sensitive to radiation damage)^1,2^. This structure dictates mass-transport and, hence, performance losses^3^, but its complexity renders three-dimensional visualization of the interactions of the catalyst layer components very challenging across the spectrum of imaging methodologies. The location of the nanosized metallic catalysts on the carbon supports was previously successfully imaged using electron tomography in a transmission electron microscopy (TEM) or scanning TEM (STEM) mode^4–7^. Electron tomography is an inherently dose-intensive technique and thus these studies omitted information on the extremely electron-beam-sensitive ionomer phase^8–10^. The poor contrast between the ionomer and carbon support^11,12^ also makes quantitative analysis a challenging computer vision problem. Ion exchange with heavy metals has been used as an enhancing preparation step for techniques based on mass-contrast such as high-angle annular dark-field STEM^12,13^ or X-ray absorption for nanocomputed tomography^14–17^. However, the nanocatalysts cannot be resolved in this case as the intensities of the different phases overlap, or for lack of resolution. Moreover, the impact on the morphology of the ionomer layers and on their interaction with radiations remains unclear^9,18^. Other studies employed fluorine chemical mapping to gain insights into the ionomer morphology within catalyst layers using either electrons^19–21^ or photons^22–25^ as ionizing radiation, but were systematically faced with trade-offs between resolution and radiation damage for access to three-dimensional data. Atomic force microscopy was recently implemented to characterize the evolution of ionomer layers during operation^26,27^, but its surface nature inherently brings limitations to imaging of buried nanocatalysts. So far, no single microscopy method has been successful in providing structural characterization of catalyst layers and quantitative analysis of the interactions of the components directly.

To achieve three-dimensional nanoimaging of catalyst layers, we implement electron tomography at cryogenic temperatures (cryo-ET) on dispersed or microtomed samples while leveraging deep learning methods for denoising and segmentation to obtain quantitative information. We start by investigating catalyst layers with graphitized supports and various ionomer contents, finding that microtomy—as a sample preparation method for three-dimensional imaging—is critical for preserving the ionomer network’s morphology. This enables the full characterization of a section of catalyst layers fabricated with porous carbon supports, in which a 3 nm highly continuous ionomer network is seen to cover 80% of the exterior carbon surface, whereas thicker ionomer morphologies such as strands and aggregates can reach 24 nm in thickness. We further differentiate between the thickness of the ionomer film on surfaces and within the network to understand the pathways of proton and gas diffusion. We also investigate the accessibility of platinum surfaces by the network and relate this to electrochemical measurements in low relative humidity conditions. Most external nanoparticles are seen to be accessible but partially covered by the network, resulting in 15% of all platinum surfaces in direct contact with the ionomer. The findings demonstrate the potential of cryo-ET for determining the morphology of catalyst layers.

## Results

### Cryo-electron tomography of catalyst layer aggregates

**Fig. 1:**
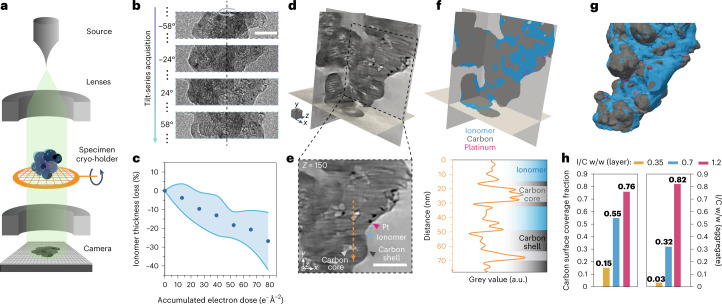
Cryo-ET workflow and analyses on Nafion–LSC–platinum aggregates. **a**, Schematic demonstrating electron tomography acquisition. Aggregates are dispersed on a grid and imaged over >70 angles in 2° increments. **b**, Representative projections acquired during the tilt series. Scale bar, 50 nm. **c**, Average beam-induced degradation measured from thickness loss of ionomer layers imaged at 98 K. Data points are the mean, whereas the shaded area represents 1 s.d. (*N* = 11). **d**,**e**, Multi-orthoslices view (**d**) and representative tomograms (**e**) of a 0.7 w/w Nafion/LSC with 8.7 wt% platinum on carbon (sample LSC7 in Supplementary Table 1). Scale bar, 50 nm; scale cube, 20^3^ nm^3^. A line profile of the grey levels is plotted along the orange dashed arrow in **e** to point out the different features. Shaded areas are provided as guides to identify regions that correspond to the ionomer phase (blue), carbon shells (dark grey) and carbon hollow cores (light grey). **f**,**g**, Segmentation results from the same aggregate (**f**) and corresponding surface rendered view (**g**). **h**, Quantitative analysis of the carbon coverage and effective I/C w/w ratio in aggregates from the catalyst layers prepared with different ionomer content (samples LSC3, LSC7 and LSC12 in Supplementary Table 1).

**Extended Data Fig. 1:**
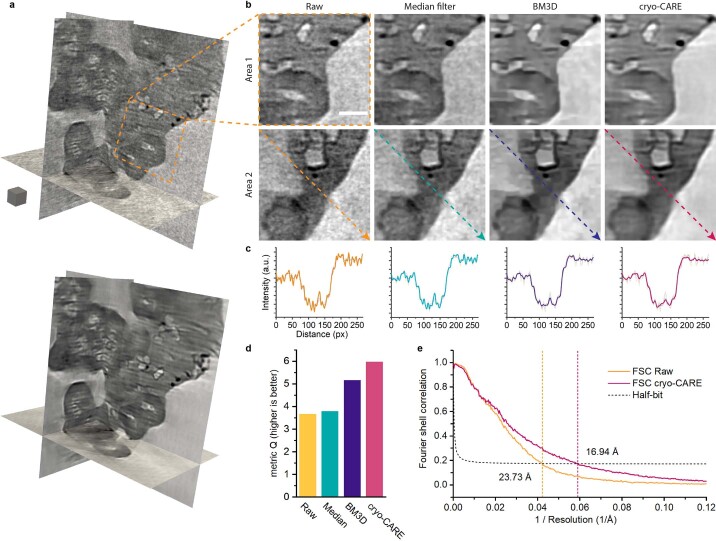
Comparison of denoising methods for tomographic reconstruction and resolution estimation. **a**, Multi-orthoslice overview of a raw reconstruction of an aggregate from the LSC7 sample and comparison with the same volume denoised with the cryo-CARE method. Scale cube is 20^3^ nm^3^. **b**, Representative areas from the reconstruction and denoising results following a median filter with a 2×2 pixels kernel, the BM3D algorithm^69,70^, and the cryo-CARE method. Median and BM3D were applied plane-by-plane while cryo-CARE is a volumetric method. Scale bar is 20 nm. **c**, Line profiles plotted from the area 2 as shown by the corresponding arrows in (**b**). **d**, Metric *Q* comparison^71^. Higher values indicate lower levels of noise and blur in 25^2^ px^2^ anisotropic patches of an image. **e**, Fourier shell correlation plots computed from the even and odd volumes before (raw) and after denoising with cryo-CARE. The resolution was estimated at the half-bit criterion.

Electron imaging of organic specimens^28,29^, including ionomers^8,9,19^, is typically performed at cryogenic temperatures because it reduces the rate of radiolysis, which is the prime source of electron beam-induced damage in such materials. To access volumetric information, we combine such an approach with electron tomography, where a series of projection images is acquired over incremented specimen tilt angles and used to compute a three-dimensional reconstruction of the specimen. A schematic of the microscope configuration operated under these conditions is depicted in Fig. 1a, and examples of bright-field TEM images of a Nafion–low surface carbon (LSC)–platinum aggregate at increasing tilt angles are shown in Fig. 1b. As demonstrated in Fig. 1c and Supplementary Fig. 1, operation at 98 K with a cumulative electron exposure of 80 e^–^ Å^–^^2^ results in a 10–40% thickness loss in the nanometre-thick layers found in the catalyst layers. To achieve a tradeoff between signal and electron beam-induced damage, a tilt series was acquired at a low accumulated electron dose of <40 e^–^ Å^–^^2^ (see Methods for further details and Supplementary Note 1 for the estimation of the minimal electron dose), thus inducing only limited degradation (see Supplementary Fig. 2). Volumetric reconstructions were then computed with the cryo-CARE method^30,31^, which integrates a denoising procedure (see the Methods for details and Supplementary Fig. 3 for the data preparation and processing pipeline). Line profiles and image quality quantification in Extended Data Fig. 1a–d demonstrates that this workflow performed much better than other methods.

In Fig. 1d,e we show the results of the reconstruction and denoising procedure on an aggregate from a catalyst layer fabricated with Nafion ionomer, LSC supports and platinum nanoparticles (sample LSC7 in Supplementary Table 1). Evaluation of the resolution using Fourier shell correlation curves (FSCs; Extended Data Fig. 1e) showed that the denoising procedure resulted in an improvement from 23.7 Å to 16.9 Å (ref. ^32^). Close inspection of the reconstruction reveals that all three phases in the aggregate can be identified on the basis of a complex combination of contrast and textural changes. As exemplified by the tomogram and line profile in Fig. 1e, the dense and highly graphitized shell of the carbon particles results in a darker contrast that surrounds a typically hollow and bright porous core. This shell was found to vary from a few carbon layers to tens of nanometres, which was further corroborated by high-resolution TEM images (Supplementary Fig. 4). Platinum nanoparticles exhibit the darkest contrast with a characteristic size of 2–5 nm. The ionomer is identified by exclusion, as a smooth, typically continuous and slightly lighter binding phase surrounding—or between—the carbon and platinum particles. Supplementary Video 1 shows animated tomographic slices in loop within 6-nm-thick sub-volumes of representative reconstructions to aid with visualization of the components.

**Extended Data Fig. 2:**
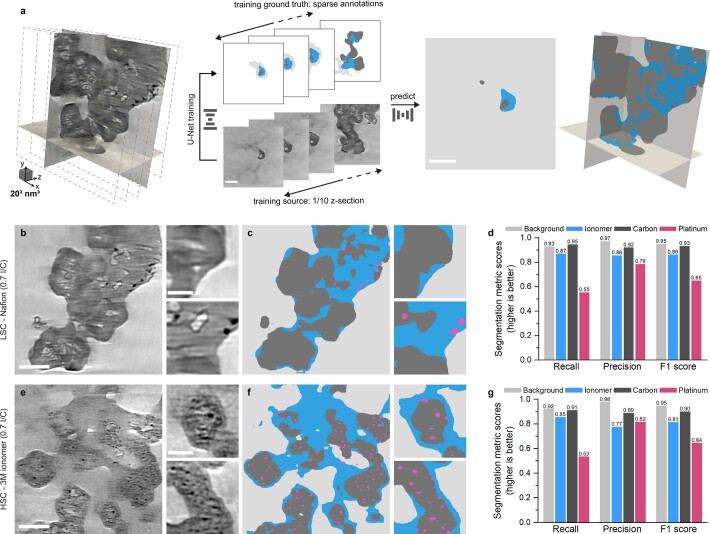
Methodology and performance of the segmentation process. **a**, Schematic depiction of the training strategy as described in Methods on the LSC7 sample. For each denoised reconstruction, 1/10 z-sections are extracted and sparsely annotated by hand in 2D. This dataset is used for training a U-Net model which is then applied to predict the segmentation maps of every z-sections, that is, plane-by-plane, in the denoised volume, before reassembling in 3D. Scale cube is 20^3^ nm^3^ and scale bars are 50 nm. **b**, **e**, Example tomograms and close-ups taken from the reconstructions and **c**, **f**, corresponding segmentation output for the LSC7 (b,c) and HSC7 (e,f) samples. Scale bars are 50 nm, 20 nm in the close-ups. **d**, **g**, Comparison of segmentation metric scores computed from a validation dataset held-out from training. For each class, recall is defined as the fraction of ground truth pixels of this class correctly labelled as such in the output, precision as the fraction of output pixels of this class correctly labelled. The F1 score incorporates both metrics in one.

Gaining quantitative insights from the reconstructions requires segmentation of the different phases. As all of the components are discernible to the human eye, this task could be performed entirely manually, but the size and complexity of the volume makes segmentation effectively intractable at scale. Automation is then desirable; but, as demonstrated by the line profile in Fig. 1e, conventional grey-level thresholding is challenged by the low intercomponent contrast. Furthermore, the remaining reconstruction artefacts can result in strong intracomponent contrast variations and areas of uncertainty. To overcome this challenge, we investigated whether deep-learning-based methods could learn from manual annotations and subsequently perform as well as a human operator in segmenting unseen data. Specifically, and for each reconstruction, we trained a U-Net convolutional neural network, whose architecture has been shown to perform particularly well on microscopy images and in data-scarce regimes^33^. Each training dataset was obtained by manually annotating one-in-ten *z*-sections, as depicted in Extended Data Fig. 2a. We note that these annotations are central to the accuracy of the segmentation pipeline and care was taken to ensure their quality. Where artefacts created ambiguities, an informed choice was made by closely inspecting the surrounding region.

After training, each model was validated by evaluating the performance against manually segmented tomograms that were unused for training (see Methods for details on the data preparation, training scheme and validation). Extended Data Fig. 2c,d and Supplementary Video 1 show that the method overall provides accurate segmentation of all components and demonstrates performance on par with human annotations on most phases. Specifically, the ionomer was segmented with a 0.86 precision, meaning that 86% of ionomer predictions from the deep learning model were similar to human ones, that is, only 14% were false positive in this volume. Applying the model to the entire reconstruction allows visualization of the segmented volumes (see Fig. 1f,g). We note that each reconstruction in this work was segmented by a unique deep learning model and that the training is not universal.

**Extended Data Fig. 3:**
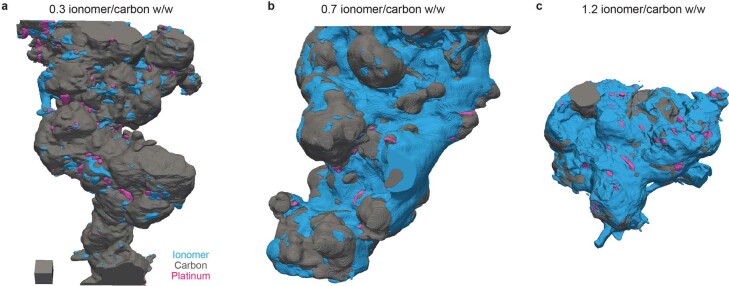
Cryo-ET reconstructions of Nafion-LSC-Pt aggregates with increasing I/C ratio. **a**-**c,** Segmented volumes of aggregates from the LSC3, 7 and 12 samples, respectively. All volumes are shown at the same scale, the scale cube is 20^3^ nm^3^.

In Fig. 1h and Extended Data Fig. 3 we demonstrate that processed reconstructions can provide qualitative and quantitative information by investigating aggregates obtained by dry-dispersion of three model catalyst layers with varying ionomer-to-carbon (I/C) weight ratios (0.35, 0.7 and 1.2 w/w; see Methods and Supplementary Table 1 for fabrication and preparation details). Volumes in Extended Data Fig. 3 allow visualization of the morphology of the aggregates and the localization of the different components with respect to each other. Furthermore, the segmented reconstructions allow computation of a range of morphological metrics characterizing the ionomer–platinum–carbon interactions (see Methods and Supplementary Note 2 for details on the calculations). With an increasing I/C ratio of the LSC electrodes, a monotonic uptrend of the local I/C weight ratio, and of the computed carbon coverage, is observed (Fig. 1h); however, the computed I/C ratio was systematically lower than expected, which we mainly attribute to disruptive effects from sample preparation by dispersion. To mitigate these effects, partial embedding^34^ and sectioning in ultramicrotomy were used in the following.

### Structure of catalyst layers

To gain insights into the morphology of a typical proton-exchange membrane fuel cell cathode, we studied a catalyst layer with 19.8 wt% platinum supported on high-surface-area carbons (HSC; porous Ketjenblack), and fabricated at a 0.7 w/w I/C ratio with a 3M 800 EW ionomer (sample HSC7 listed in Supplementary Table 1). Sample preparation with microtomy was found to maintain a well-preserved open porosity while being amenable to tomography (as illustrated by the tilt series in Supplementary Video 2). Fourier shell correlation curves indicate a 17.5 Å resolution in the reconstructed volume.

Segmentation was performed following the same methodology established on LSC samples. In this case, the outermost, pseudo-graphitic layers of the carbons were usually well evidenced due to the greater spacing of interlayers compared with the graphitized LSCs. Furthermore, the internal porosity causes textural variations in the carbons, as opposed to the ionomer, which was typically smoother. Side-by-side comparisons between greyscale tomograms and segmentation results are presented in Supplementary Video 1 and Extended Data Fig. 2e,f. There again, the deep learning model trained for this volume was found to perform well compared to manual annotations as shown in Extended Data Fig. 2g. Performance metrics were found to be close to the ones computed on the LSC7 sample, demonstrating the reproducibility of the method in matching human performance across different samples. Energy dispersive spectroscopy (EDS) analysis on the site of the tomographic reconstruction was also performed, as detailed in Supplementary Fig. 5. We find that signals associated with the ionomer can be seen throughout the layer and overall match with the segmentation results, despite limitations in imaging thin ionomer layers with EDS (further discussed in Supplementary Note 3).

**Fig. 2:**
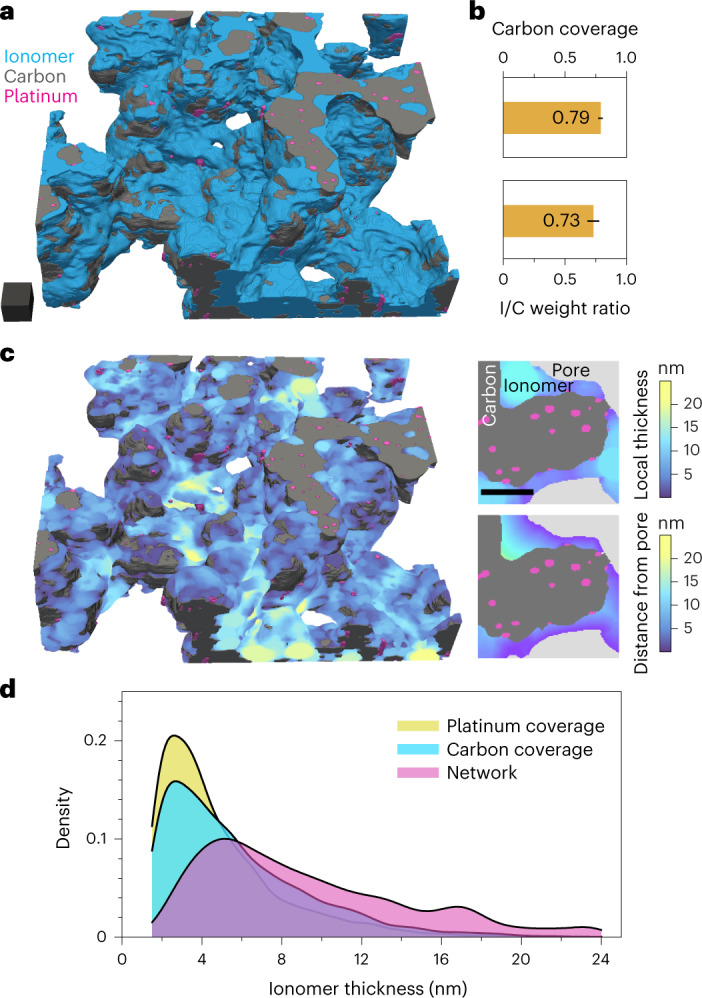
Cryo-ET reconstruction of a microtomed 3M ionomer–HSC–platinum catalyst layer and ionomer network analysis. The catalyst layer was prepared with a 0.7 I/C weight ratio and a 19.8 wt%_Pt_ catalyst (sample HSC7 in Supplementary Table 1). **a**,**b**, Segmented reconstruction (**a**) and measurements of I/C weight ratio and carbon surface coverage (**b**). Error bars represent the measurement error due to segmentation (see Methods). Scale cube, 20^3^ nm^3^. **c**, A 3D map of the ionomer local thickness, and magnified images illustrating the difference in calculation to the local thickness algorithm, and a graph-based distance calculation from the external pore. Scale bar, 20 nm. **d**, Distribution of the ionomer thickness plotted for different sampling and calculation methods. The network thickness is sampled randomly throughout the ionomer in the local thickness map, whereas carbon and platinum coverages are sampled randomly at their respective interfaces with the ionomer in the distance-from-pore maps. The cutoff at 1.5 nm accounts for the resolution limit.

A surface rendering of the segmented reconstruction is presented in Fig. 2a, and an animated visualization of the volume is available in Supplementary Video 3. Relatively small and oriented carbon aggregates—formed of primary beads 20–40 nm in diameter—are observed, similar to the structures seen in TEM images of dispersions of the Pt/C catalyst (some examples of which are shown in Supplementary Fig. 6). The external surface area of the primary carbon particles is calculated to be $120 \, \mathrm{m}^2 \, {\mathrm{g}}^{-1}_{\mathrm{carbon}}$, not accounting for the internal porosity of the HSCs, which is in the range of typical solid, non-porous carbon supports^35^. Internal platinum nanoparticles and some carbon pores can be visualized in the reconstruction (Supplementary Fig. 7), but a substantial 50% of the surface fraction of this porosity is known to be held in pores smaller than the resolution of our data^36^; pore analysis was thus excluded from this work. The ionomer network is visible as strands and thick patches connecting carbon aggregates and we calculate an I/C weight ratio of 0.73 (see Fig. 2b), which is in excellent agreement with the bulk value of 0.7. In comparison with results from dispersions in Fig. 1, this demonstrates the importance of adequate sample preparation for analysis of the ionomer phase.

We then studied the ionomer network morphology—an important characteristic of catalyst layers linked to reactant mass transport resistances and fuel cell performance^37–39^. A connected components analysis of the ionomer phase (Supplementary Fig. 8) indicates high connectivity with the single largest component accounting for 99.4% of the ionomer volume. Coverage of the external carbon surfaces is found to reach 78% in this area, indicating that at this ionomer content, the majority of exterior carbon surfaces are in contact with the ionomer phase. To investigate the thickness and homogeneity of the network, we first computed the ionomer network thickness distribution using the local thickness algorithm^40^, which accounts for the thicker areas and strands, and relates therefore to proton transport within the network. We compare this with the carbon coverage and platinum coverage thickness distributions, obtained by calculating the distance from their respective surface to the nearest pore between the primary carbon particles (see Methods for details) which pertain more directly to oxygen transfer through the ionomer film. A colour-coded 3D map of the local thickness is presented in Fig. 2c, alongside with magnified sections taken from the volume and illustrating the difference between the two calculation strategies. The corresponding distribution plots are shown in Fig. 2d. We find a wide range of ionomer thicknesses, as observed previously from 2D micrographs^41^ and hybrid simulation–observation methods^14^, with the network being on average 9.2 nm thick. The map and distribution also demonstrate the presence of large ionomer areas, up to 24 nm thick. Interestingly, complementary results obtained with ion-exchange (and discussed in more detail in Supplementary Note 4 and Supplementary Fig. 12) indicate that phase separation may exist within these large agglomerates of pure ionomer. In comparison, the platinum and carbon coverage extracted data exhibit narrower and close distributions, with mean thicknesses of 4.9 and 5.7 nm, respectively, and a mode centred around 3 nm in both cases. This comes close to a geometrical estimation of the average ionomer thickness of 3.8 nm at 0.7 w/w I/C, $120 \, {\mathrm{m}}^2 \, {\mathrm{g}}^{-1}_{\mathrm{carbon}}$ and 78% coverage on a hypothetically planar carbon surface, with a higher value anticipated for results from tomography due to the more complex geometry. Furthermore, inspection of bright-field TEM images of the same microtomed sample (Supplementary Fig. 9a–e) demonstrates that similar ionomer morphologies are observed across the section, whereas measurements of the thickness of the ionomer coverage are closely matching the tomography results (Supplementary Fig. 9f).

To further study the representativeness of these results, the I/C weight ratio, carbon coverage fraction and carbon coverage thickness were computed on three sub-areas from the volume used in Fig. 2. The results are plotted in Supplementary Fig. 10. The I/C weight ratio was found to considerably differ in the sub-areas, which is coherent with the few large ionomer agglomerates accounting for a substantial fraction of the ionomer volume. The carbon surface coverage and coverage thickness were found to closely match the measurements that included the entire volume. Furthermore, we performed EDS analysis of the microtomed section (Supplementary Fig. 11), which indicates that, at 1*σ*, the platinum-normalized fluorine signal varies by less than 8.4% from its mean over areas of 0.5^2^ µm^2^. Under the assumption that platinum is well distributed in the electrode, this suggests that ionomer distribution throughout the layer was relatively homogeneous. Taken together, these results suggest that analyses performed at the scale accessible in electron tomography can be considered representative.

In comparison with other attempts to characterize the ionomer film thickness in catalyst layers, we find results that are coherent with Cetinbas and co-workers^14^ at this ionomer content, but values much smaller than reported previously from STEM tomography^12^ and in situ atomic force microscopy^27^. These discrepancies with former studies could arise due to the difference in sample preparation (see Supplementary Note 4 on ion-exchange for contrast enhancement), and due to the hydrated state of the ionomer and a possibly higher ionomer content. Our measurements nonetheless form a coherent picture with the morphology of ionomer dispersions that are known to exhibit primary rod-like aggregates with radii of 1.5 to 2.5 nm depending on the equivalent weight and side-chain length^42,43^, and secondary ones up to hundreds of nanometres depending mainly on the dispersion environment^44,45^. This agreement suggests that, at this ionomer content, the carbon and platinum surfaces would be most frequently covered by a single primary ionomer aggregate, and, therefore, that the state of the ionomers in dispersion would be strongly linked with the final morphology in catalyst layers.

**Fig. 3:**
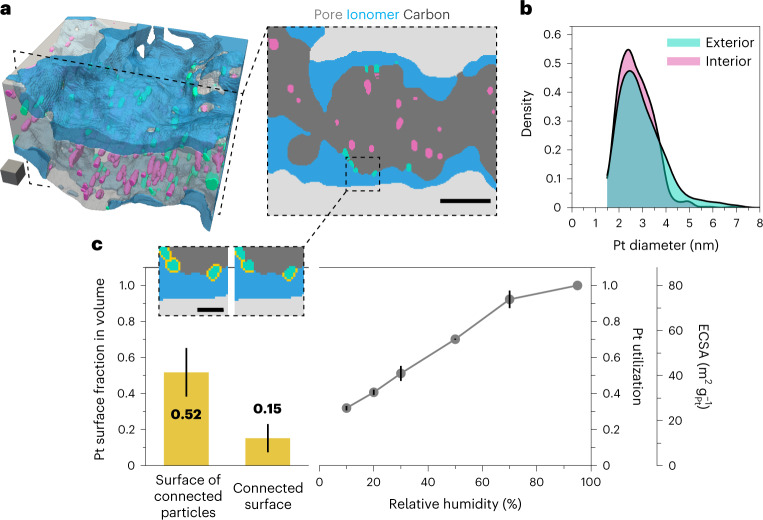
Platinum-related morphology analysis in the microtomed 3M ionomer–HSC–platinum catalyst layer. **a**, Sub-volume and magnified 2D maps from the segmented reconstruction in Fig. 2 demonstrating the localization of platinum nanoparticles interior (pink) or exterior (green) to the porous carbon supports. Scale cube, 10^3^ nm^3^; scale bar, 20 nm. **b**, Distribution of the platinum diameters as a function of their location. The cutoff at 1.5 nm accounts for the resolution limit. **c**, Comparison of platinum surface measurements from tomography and electrochemistry. As illustrated in the magnified slices (scale bar, 5 nm), the measurements from the tomographic reconstruction represent the fraction of the platinum surface in direct contact with the ionomer (connected surface), and the fraction of the platinum surface of all particles in contact with the ionomer (surface of connected particles). Electrochemical measurements are the ECSA and platinum utilization from CO stripping surface area measurements as a function of relative humidity in the membrane electrode assembly (MEA), scanned from 0.1–1.0 V at 10 mV s^–1^ and 80 °C. The grey dots represent the average of N = 2 measurements. Error bars represent the measurements error due to segmentation for tomography (see Methods) and the min–max values of *N* = 2 measurements for electrochemical data.

The reconstructed volume further allows study of the morphology and interactions of the platinum nanoparticles with the carbon support and ionomer network. Figure 3a shows a three-dimensional visualization and a close-up tile of a sub-volume taken from the reconstruction in Fig. 2. A substantial fraction (46%) of the nanoparticles are found to reside in the nanopores within the carbon primary particles, in agreement with previous work on this type of high-surface-area carbons^4,5^. The distributions of interior and exterior platinum sizes are depicted in Fig. 3b. Both populations exhibit similar morphologies, with an average diameter of 2.7 and 3.0 nm for the interior and exterior particles, respectively, and a slightly wider distribution for the exterior particles (*σ*_interior_ = 0.7 nm, *σ*_exterior_ = 1.0 nm), probably due to particle growth constrained by the inner porosity of the carbon support during synthesis.

Focusing on the exterior platinum particles and their connectivity to the ionomer network, we then compared surface areas that were computed from the tomograms to bulk measurements. Figure 3c shows that different quantities can be obtained from tomography. As depicted by the magnified slices in Fig. 3c, the connected surface is defined as the platinum surface in direct contact with the ionomer, which accounted for 15 ± 8% of the total platinum surface in the reconstruction. By comparison, the surface of connected particles is the measure of the total surface of all particles in contact with the ionomer, and was 52 ± 13% of the total platinum surface in the reconstruction. The discrepancy between these quantities can be partly attributed to a tendency in the segmentation results to embed platinum more deeply than would be realistic, which is captured by our error estimation from mean absolute error calculations with the evaluation dataset (see Methods for details). For comparison with electrochemical measurements, we measured the electrochemical surface area (ECSA) by CO stripping at varying relative humidity values—a method routinely used to assess platinum utilization^46,47^. As oxidation of CO requires water and proton-conducting pathways, measurement of the ECSA at high relative humidity provides information on all platinum surfaces within the layer due to condensation of water on the carbon surface and within the carbon nanopores. Conversely, measurements at low relative humidity would exclude surfaces that are not connected to the ionomer phase, which contains water and conducts protons even in dry conditions. The plot shown in Fig. 3c demonstrates the decrease in platinum utilization (the ratio of ECSA at a given relative humidity to the measurement at 95% relative humidity) as measurements are performed at lower relative humidity. We note that the measurements do not reach a plateau within the investigated range, an observation that is further discussed in Supplementary Note 5. The ECSA at 95% relative humidity averages $79.9 \, {\mathrm{m}}^2 \, {\mathrm{g}}_{\mathrm{Pt}}^{-1}$, which correlates well with the $76.2 \, {\mathrm{m}}^2 \, {\mathrm{g}}_{\mathrm{Pt}}^{-1}$ total surface area from tomography and indicates that all platinum is accessible for CO stripping at these high relative humidity levels^4^, including platinum particles buried within the nanopores of the primary carbon particles. At 10% relative humidity, CO stripping measurements indicate a platinum utilization of 31%, which lies in between the values of the connected surface and of the surface of the connected particles computed from tomography. This suggests that a larger area than the one in direct contact with the ionomer participates in the ECSA measurements even at low relative humidity. As further discussed in Supplementary Note 5, this could indicate remaining interfacial and bound water^48^, or proton surface diffusion^49^ in CO stripping measurements at low relative humidity.

## Discussion

In summary, we elaborated on an approach that provides access to quantitative, three-dimensional data at nanometre resolution, allowing a detailed observation of the proton-exchange membrane fuel cell catalyst layer structure with all of its components. It was demonstrated that—although not completely suppressing electron-beam-induced damage to the ionomer—performing cryogenic electron tomography sufficiently mitigates these effects and allows for volumetric reconstruction. Furthermore, advanced image processing methods enabled quantitative measurements of all of the components, whereas sample preparation with ultramicrotomy allowed observation of a highly continuous network and study of its characteristics. We confirmed that a wide range of ionomer thicknesses exist within the layer (up to 20–24 nm), with the majority of the platinum particles on the exterior of the carbon supports being covered by single ionomer aggregates, 3–4 nm thick. Furthermore, the high coverage of the external carbon surfaces results in the ionomer network connecting the majority of exterior platinum nanoparticles on porous high-surface area carbon supports.

More generally, routine investigations of catalyst layers with cryo-ET requires further technique advancements. In this work we relied extensively on human operations while leveraging machine learning as an extension to manual segmentation. This is a laborious process, which, in combination with the remaining reconstruction artefacts and noise, can entail a degree of uncertainty. Nevertheless, our results were systematically corroborated by bulk sample measurements and properties, including the I/C ratio, ionomer average thickness and platinum specific area. Looking forward, to limit uncertainty and streamline the image processing workflow, methods for improving the signal-to-noise ratio in the acquisitions are imperative. Envisioned enhancements could leverage improvements in instrumentation, such as more sensitive cameras and phase plates. In addition, strategies for reducing manual input in the segmentation procedure could build on a generic model trained on synthetic images that would be fine-tuned to each reconstruction requiring only a few annotations.

Our results therefore pave the way towards comparison of different catalyst layer morphologies at the nanoscale. Further investigations building on this approach could offer valuable insights into the effects of carbon–ionomer interface engineering, which have been recently implemented to develop improved carbon coverage^37,38^, and could help unravel the relationships between the state of ionomer in dispersions and catalyst slurries, and their final film and network morphology in catalyst layers.

## Methods

### Materials

Samples used in this study were Pt/C catalyst layers prepared by Mayer rod-coating catalyst slurries onto PTFE sheets. All characteristics of the catalyst layers are summarized in Supplementary Table 1. Two carbon black support types were used: a compact, low-surface-area graphitized Vulcan-type carbon (LSC) and a high-surface-area, porous Ketjenblack (HSC; both were supplied by Tanaka Kikinzoku Kogyo). For the LSC carbon, platinum was deposited via a polyol reduction method^50^ at a loading of 8.7 wt%, quantified by thermogravimetric analysis. Three catalyst layers were prepared with those, using Nafion D2021 and I/C ratios of 0.35, 0.7 and 1.2. For the HSC-based catalyst, the as-received material contained 19.8 wt% platinum that had been deposited by the manufacturer, and a catalyst layer was prepared using a 3M 800 EW ionomer (where EW represents the equivalent weight in units of ${{\mathrm{g}}_{\mathrm{ionomer}}} \, {\mathrm{mol}^{-1}}_{\mathrm{SO}_{3}{\mathrm{H}}}$) at I/C 0.7. The choice of using a lower equivalent weight ionomer was found to improve coating homogeneity, as seen from a backlight observation of the layers.

### Sample preparation for electron microscopy

Samples for TEM were prepared by dry-dispersion (samples LSC3, LSC7 and LSC12) or ultramicrotomy (HSC7). For the dispersed samples, stripes of a catalyst layer were scraped with a scalpel blade and the resulting powder was lightly crushed between two glass slides to further break up the larger clumps. TEM grids—typically lacey carbon, 200 mesh (Electron Microscopy Sciences)—were repeatedly placed onto the powder until enough aggregates had been transferred, as observed from optical microscopy. Microtomed cross-sections were prepared with partial or full embedding of small triangular stripes cut from the catalyst layers, as described in refs. ^34,51^. For partial embedding, the first resin layer was a pre-cured Gatan’s G2 and the Embed 812 epoxy (Electron Microscopy Sciences)—cured at 60 °C overnight—was used to form the blocks. Microtomy was performed at room temperature on a Leica EM UC7, using a Diatome cryo 35° diamond knife with a boat; 100–150 nm thin sections were cut and floated on water before transferring to TEM grids.

### Radiation damage evaluation and tilt-series acquisition

Tilt- and dose-series were acquired on a ThermoFisher Scientific F20 equipped with a Falcon III direct electron camera (ThermoFisher Scientific), and operated at 200 kV in bright-field mode and using a 40 µm objective aperture. The sample holder was a Gatan 914. When working in cryogenic conditions the holder was cooled in-column to −175 °C (98 K) and contamination by ice growth was regularly controlled, with an operating window usually lasting 3–5 h for each grid, depending on humidity conditions.

For radiation damage evaluation, dose-series of microtomed samples from the HSC7 sample were acquired at a dose of 1,300 e^–^ nm^−2^ per image, with an initial 120 e^–^ nm^−2^ required for positioning and focusing. The thickness of the ionomer coverage was manually measured as a function of the accumulated dose in 11 positions, with initial thicknesses ranging from 3 to 7.5 nm. The plots were linearly fitted, and the dose corrected to ensure an intercept of zero.

For tomographic acquisition, tilt-series of 4,096 × 4,096 pixels images were acquired at 50 kx magnification, resulting in a pixel size of 0.2 nm. ThermoFisher Scientific’s TEM tomography software was used to acquire images as sequences of 30 frames (each 50 ms) totalling 1.5 s of exposure time per angle. The electron dose rate was typically 20–33 e^–^ nm^–^^2^ s^–^^1^, that is, 30–50 e^–^ nm^–^^2^ per angle. On average, an initial irradiation with a dose of 100 e^–^ nm^–^^2^ was required to position the region of interest, optimize the focus and obtain a snapshot of the initial state before acquisition. Each tilt-series then consisted of 65–75 projections in 2° increments. The dose rates, total accumulated dose and sampling range for each acquisition are detailed in Supplementary Table 2.

### Tomography reconstruction and image processing

Before reconstruction, frames at each angle were aligned using the MotionCor2 software^52^ and split in two interleaved even/odd stacks, each containing half of the frames. Frames were summed to a single image and 2 × 2 binned; the procedure was repeated for all of the angles before assembling in two tilt-series image stacks. We found that the combination of low signal-to-noise ratio, bright-field mode, lacey carbon grids and microtomed cross-sections resulted in automated alignment methods such as centre-of-mass, cross-correlation or even IMOD’s marker tracking performing poorly. Consequently, alignments were performed semi-manually using a platinum nanoparticle visible at as many angles as possible, and a tool for the FIJI software written in-house in ImageJ macro language^53,54^. Briefly, the macro allows users to place markers tracking a feature of interest throughout the tilt-series, and subsequently translates the images to align these markers. Shifts can be recorded and applied to other stacks, so that the exact same alignments were done to the even and odd acquisition series. Tilt-series alignments were further refined manually down to pixel accuracy using the Tomviz software^55^. Finally, the rotation axis was identified by tracking the trajectories of nanoparticles throughout the tilt-series and corrected for centre and orthogonality.

Tomographic reconstruction was performed using the Astra toolbox^56,57^ with code implemented in Python. The cuda version of the simultaneous iterative reconstruction technique with 20 to 30 iterations was used to reconstruct two volumes from the even and odd tilt-series. Volumes were then used to train a deep learning model for denoising in a Noise2Noise regime^58^. Our code made heavy use of the open-source notebooks created by Bucholtz et al. for the cryo-CARE workflow and we therefore refer the reader to the relevant publications^30,59^ and GitHub repository^60^. A summary of the workflow is shown in Supplementary Fig. 3. Parameters were identical to those used by the authors and training was run typically for 150–200 epochs. The model was then applied to both even and odd volumes, before summing the output predictions into the final reconstruction.

Segmentation was performed using the YaPiC Python toolbox^61^. The platform performs as a pixel classifier, allowing to train deep learning models from sparse annotations. For each reconstruction, a training dataset was generated by sampling one in ten z-sections throughout the volumes. Each section was annotated manually using the QuPath software^62^ to obtain ground truth data. An example is shown in Extended Data Fig. 2, along with a schematic depiction of the method. For the annotation procedure, all components could be visually identified, following features described herein. However, reconstruction artefacts, the missing wedge elongation and remaining noise occasionally challenged this task. In such cases, close inspection of the surrounding volume allowed to make an informed decision.

The neural network used for segmentation had the classic U-Net (2D) architecture^33^, and minibatch-wise normalization, data augmentation by flipping, 20% validation and 50 training steps per epochs were used. Training was typically run for 500 epochs. We chose to work with segmentation in 2D rather than 3D for ease of annotation and speed of training, as 3D models have considerably more parameters to be optimized, which increases the risk of overfitting in our data-scarce regime. After training, the performance was evaluated on the training dataset and the annotations were completed as necessary to fine tune the models, before applying them to the entire reconstructed volumes. The models have a soft-max activation as the final layer, so that each pixel is returned as a vector whose elements are the scores associated with each class and have been normalized between 0 and 1, resulting in a probability-like map per class. Consequently, each map was binarized using Otsu’s method^63^ and the resulting volumes were reduced to a single stack with a singular grey level per class. Finally, a median filter with a kernel size 1 x 1 x 4 voxels was applied to account for the anisotropy of the 2D segmentation.

### Resolution estimation

Resolution of the reconstruction before and after denoising was estimated using FSCs between the volumes reconstructed from the even and odd stacks. Computation was performed using the FSC program from Image Science^64^. The resolution was estimated at the half-bit criterion.

### Data analysis

From the segmented volumes, a range of metrics was computed, including volume fractions, carbon surface area and coverage by ionomer, connected components, ionomer network thickness, size distribution of the platinum nanoparticles and their accessibility and surface area. All operations and measurements were performed with FIJI and the MorpholibJ^65^ and BoneJ2^66,67^ plugins or in Python using mainly the scikit-image, SciPy and PoreSpy^68^ libraries. Volumes and multi-orthoslice views were rendered with Tomviz^55^. Details on the methodology and software used for each metric are given in Supplementary Note 2.

Where applicable, an error measurement was estimated by calculating the mean absolute error between measurements from the processed volume and from an evaluation dataset, which was a set of densely and manually annotated tiles that were not used for training.

### EDS analysis

Energy dispersive spectroscopy hypermaps were acquired on a ThermoFisher Scientific Osiris TEM operated at 200 kV at room temperature, with samples prepared by ultramicrotomy. For the fluorine distribution at the catalyst layer level, the F Kα and Pt Lα and β lines were fit to obtain net counts maps with a 50^2^ nm^2^ pixel size. The fluorine to platinum weight fraction was calculated from k-factor quantification performed in Velox (ThermoFisher Scientific). Under the assumption that platinum distribution is homogeneous throughout the electrode, the resulting map is representative of the ionomer distribution. For statistics, the mean fluorine weight fraction was measured in sub-areas of 500^2^ nm^2^ tiled over the entire acquisition. For the analysis of the HSC7 tilt-series acquisition site, a map with 0.62^2^ nm^2^ pixels was acquired with a 0.6 nA probe and a resulting total accumulated dose of 4.9 × 10^7^ e^–^ nm^–^^2^. For net counts, the maps were processed with the Velox software again, with the F Kα, C Kα and Pt Lα and β lines fitted after a 3*σ* gaussian pre-filter. Decomposition was performed with the Hyperspy Python toolbox, after a 4x spatial binning preprocessing step and using the non-negative matrix factorization algorithm. To prevent interference from silicon contamination (see discussion in Supplementary Note 3), the Si Kα was masked before the operation.

### Electrochemical measurements

Membrane electrode assemblies with an active area of 5 cm^2^ were prepared using the HSC7 catalyst layers as cathodes, whereas anodes were fabricated based on a commercial 20 wt% Pt/Vulcan catalyst (TEC10V20E, Tanaka Kikinzoku Kogyo). The MEAs were prepared by hot-pressing anode/cathode decals at 155 °C for 3 min onto a 15 µm reinforced membrane. Platinum loadings were determined by weighing decals before and after hot-pressing.

Fuel cell measurements were performed in a stainless-steel single-cell hardware (Fuel Cell Technologies) fitted with 5 cm^2^ active area graphite flow fields (0.5 mm wide channels and lands, Poco Graphite). For the anode and cathode, Freudenberg H14C10 was used as a diffusion medium with a compression of 14%. Testing was performed on an automated Greenlight G60 test station. All MEAs were conditioned before electrochemical characterization using a voltage-controlled break-in procedure (H_2_/air at 1,390/3,320 nccm, 80 °C, 100% relative humidity, and 150 kPa_abs inlet_), following a sequence of 0.6 V (45 min), open circuit voltage (5 min) and 0.85 V (10 min) for ten cycles.

The platinum utilization was then determined by CO stripping voltammetry at 80 °C and relative humidity of 10%, 20%, 30%, 50%, 70% and 95%. Briefly, the cathode compartment, equilibrated to a set temperature and relative humidity, was flushed with CO (10 vol%, balance N_2_) for 10 min, followed by an N_2_ purge of the entire test station and its gas supply. Three consecutive cyclic voltammograms were recorded by scanning the electrode potential between 0.1–1.0 V_RHE_ at 10 mV s^−^^1^. The charge corresponding to CO electro oxidation—apparent in the first anodic scan at potentials above 0.6 V_RHE_—was obtained by integrating the current using the second anodic scan as a baseline, where no further CO oxidation occurred. The integrated charge can be converted into an electrochemically active platinum surface area using a charge density of ${{420 \, \upmu {\mathrm{C}} \,{\mathrm{cm}}^{-2}_{\mathrm{Pt}}}}$ for linearly bound CO. Finally, the relative humidity-dependent platinum utilization is calculated by dividing the electrochemically active platinum surface at a given relative humidity by its pendant at full humidification, that is, the electrochemically accessible platinum surface area at 95% relative humidity.

## Supplementary information

Supplementary Information Supplementary Notes 1–5, Figs. 1–12, and Tables 1 and 2.

Supplementary Video 1 Comparison of animated grayscale reconstructions and segmentation results.

Supplementary Video 2 Aligned tilt series of the HSC7 sample.

Supplementary Video 3 Animated view of the HSC7 segmented reconstruction.

## Data Availability

The data and analyses for the figures in the main text are available at 10.5281/zenodo.7730078. All of the data in the study are available from the corresponding authors on reasonable request.

## Code availability

The code and scripts developed and used for this study are available at 10.5281/zenodo.7730078.
