# Supplementary material for: Three-dimensional nanoimaging of fuel cell catalyst layers
Source: Nat Catal. 2023 Apr 17;6(5):383–91. doi: 10.1038/s41929-023-00947-y (PMC10212762; doi:10.1038/s41929-023-00947-y)
Supplement: Supplementary file 1 — Supplementary Notes 1–5, Figs. 1–12, and Tables 1 and 2. [file 41929_2023_947_MOESM1_ESM.pdf]

nature catalysis

Article https://doi.org/10.1038/s41929-023-00947-y

Three-dimensional nanoimaging of fuel cell catalyst layers

In the format provided by the authors and unedited

Content

Supplementary Notes **2**
Supplementary Note 1: minimal electron dose for complete tilt-series 2
Supplementary Note 2: detailed methods for data analysis 2
Supplementary Note 3: EDS analysis on the site of the HSC7 reconstruction 4
Supplementary Note 4: ion-exchange of ionomer layers for enhanced mass contrast 4
Supplementary Note 5: Pt utilization in low RH conditions 5
Supplementary Figures **7**
Supplementary Figure 1: dose-series 7
Supplementary Figure 2: e-beam induced damages during tilt-series acquisition 7
Supplementary Figure 3: workflow of cryo-CARE denoising 8
Supplementary Figure 4: morphology of graphitized Vulcans 9
Supplementary Figure 5: EDS on HSC7 site 10
Supplementary Figure 6: morphology of Ketjenblacks aggregates 11
Supplementary Figure 7: pores in Ketjenblacks 11
Supplementary Figure 8: connected components analysis of the ionomer network 11
Supplementary Figure 9: ionomer morphologies in HSC7 catalyst layer 12
Supplementary Figure 10: representativeness of measurements within HSC7 volume 12
Supplementary Figure 11: electrode homogeneity 13
Supplementary Figure 12: ionomer morphology after ion-exchange 14
Supplementary Tables **15**
Supplementary Table 1: composition of various catalyst layers 15
Supplementary Table 2: tilt-series acquisition parameters 15
Supplementary References **16**

Supplementary Notes

Supplementary Note 1: minimal electron dose for complete tilt-series

To estimate the minimal electron dose that could be used for tilt-series acquisition, we considered that the most critical step for the final resolution was the manual alignment procedure, in which at least one Pt nanoparticle had to be identifiable at all tilt angles with adequate resolution and contrast. When operating in a dose-limited regime, Egerton proposed that the resolution is governed by the following equation[1]:

$$\delta = (SnR) \times (DQE)^{-1/2} \times F^{-1/2} \times D_e^{-1/2} \times \frac{\sqrt{2}}{|C|} \quad (1)$$

So that

$$D_e = \frac{2}{DQE \times F} \times \left(\frac{SnR}{\delta \times C}\right)^2 \quad (2)$$

Where, D_e is the electron dose, DQE the detector quantum efficiency, F a signal efficiency relating the number of recorded electrons to the fluence required to generate them, SnR the signal-to-noise ratio, δ the (dose-limited) resolution, and C the Weber contrast of the feature relative to the adjacent background.

In a first approximation, we considered a 2 nm resolution requirement for accurate localization of an average Pt nanoparticle in a Pt/C catalyst, with a Rose criterion for the SnR of 3. In our defocused phase contrast imaging conditions, we measured a Weber contrast ~ 0.5, and used a signal efficiency of 0.87, as proposed by Egerton[1]. The DQE of our camera had been previously measured to be 0.46 at half the Nyquist frequency.

Introducing these values in equation (2) gives a minimal electron dose requirement of 44 $e^{-}.nm^{-2}$, so that we have typically operated in a range of a few tens of $e^{-}.nm^{-2}$ per image in the tilt-series acquisitions, as detailed in the methods. In effect, it was observed that these conditions allowed for manual alignment of about half of the acquired tilt-series and that the task remained highly challenging, qualitatively confirming that this dose is close to the lower operable limit.

Supplementary Note 2: detailed methods for data analysis

All operations and measurements for the following metrics were performed with FIJI and the MorpholibJ[3] and BoneJ2[4,5] plugins or in python using mainly the scikit-image, SciPy and PoreSpy[6] libraries.

Volume and mass fractions, carbon surface area: volumes and surfaces of the binary volumes were calculated with MorpholibJ, and related to mass fractions and surface assuming an ionomer and carbon density of 2000 $kg.m^{-3}$.

Carbon surface coverage: voxels at the carbon interfaces were identified by creating a mask of the carbon phase dilated by one voxel, then subtracting the original carbon phase from it. This edge mask was used to sort carbon-neighboring voxels as a function of their value, i.e., their class. The surface coverage was computed as the fraction of edge voxels belonging to the ionomer phase, over all edges.

Connected components: The connected component analysis was performed using the component labeling tool of the MorpholibJ plugin with a lower volume threshold at 1.5^3 nm^3 to account for limitations in resolution.

Ionomer network thickness: The ionomer network thickness was computed with the local thickness algorithm implemented in PoreSpy, which returns for each voxel the diameter of the biggest sphere inscribed within the phase while containing the voxel. To plot a distribution, 10000 points were randomly sampled within the ionomer phase.

Pt and carbon ionomer coverage thickness: Following Cetinbas *et al.*[7], the coverage thickness was computed using a graph-based approach to calculate the geodesic distance between a sample of points at the Pt or carbon surface and the closest pore exterior to the primary carbon particles. Briefly, an adjacency matrix was created from the ionomer phase, where each voxel was connected to its 26 neighbors by edges whose weight were their Euclidian distance. Dijkstra's algorithm was then repeatedly used to search the shortest path between all voxels of the ionomer phase and any voxel of the ionomer / pore interface, effectively creating a distance-from-pore map. To make the computation tractable within reasonable time, the volume was subdivided in 200^3 vx^3 patches that were subsequently reassembled in a single map after distance calculations. Distributions were obtained by sampling 10000 points at the surface of Pt or carbon within this map.

Pt size distribution: Prior to analysis, Pt nanoparticles contacting each other were separated and individually labeled using a distance transform watershed segmentation. The particles were then fitted with ellipsoids, and diameters were calculated by averaging the intermediate and minor diameters of the ellipsoids in order to account for missing wedge elongation that artificially increases the major diameters. The distributions were truncated below 1.5^3 nm^3 to account for the resolution.

Pt position and accessibility: For each platinum nanoparticle, the number of contacting voxel with the background or the ionomer was first quantified. Particles were classified as exterior if their edges contacted another phase than carbon, and further defined as connected if contacting the ionomer phase. As described above, we restrict the definition of contact to areas above 1.5^2 nm^2 to account for the resolution. For surface fractions, the total contact areas (with background or ionomer) were summed and divided by the total Pt area. The surface fraction of the connected particles was computed similarly, taking into account the entire surface of all connected particles as depicted in Fig. 3a of the main manuscript

Supplementary Note 3: EDS analysis on the site of the HSC7 reconstruction

EDS analysis was carried out to confirm the ionomer presence in the area reconstructed in Fig. 2 of the main text. The analysis was done on a different microscope and, while the precise location could be found, changes were apparent between the images at the end of the tilt-series acquisition and those prior to the EDS analysis. Clear material displacement (e.g., bottom-left corner of the image in Supplementary Fig. 5b) and overall shrinkage can be seen in Supplementary Fig. 5b-c . This is likely due to the abrupt thawing and condensation/deposition the sample/grid was subjected to when removing the cryo-holder from the column, and/or to beam-induced damage during the first positioning frames prior to the EDS analysis, which was performed at RT and relatively high dose (see Methods). However, the majority of the tilt series area was intact and could be compared to the EDS results.

As seen in the integrated EDS spectrum depicted in Supplementary Fig. 5e and e, the fluorine signal was overall low in this area. This could be attributed to degradation during the positioning frames and, possibly, to F-losses during tilt-series acquisition that were not apparent in the images (as seen in Supplementary Fig. 2f). In addition, the area suffered strongly from carbon buildup during EDS acquisition, which could arise from contamination during condensation/deposition on the cold grid, and this also limited the acquisition time and SNR. Finally, based on previous studies[2], it is uncertain that a 2-3 nm-thick ionomer layer can generate enough counts before most of the fluorine is lost.

We note that the whole sample/grid suffers from Si contamination (Fig. 1e), which was not detected in previous EDS on this catalyst layer, as shown for example in Supplementary Fig. 11. Upon further investigation, we have found it to be often present when re-acquiring data on year(s)-old samples, as was the case for this analysis. In comparison the data in Supplementary Fig. 11 was acquired on a grid that had not been stored for such a long time. Therefore, this Si contamination most likely originates from the long-term storage in non-inert conditions inside typical grid-storage boxes.

To improve the SNR, we also applied multivariate statistical analysis by way of decomposition with non-negative matrix factorization. Results are displayed in Supplementary Fig. 5i-k, and show that the identified components have compositions than can be related to the Pt catalyst, the ionomer and the carbon supports, and that marginal improvements are gained in the SNR.

Comparing the projected segmentation results with the EDS data therefore demonstrate that ionomer is present throughout the area and in locations agreeing the with tomography results.

Supplementary Note 4: ion-exchange of ionomer layers for enhanced mass contrast

One of the difficulties of resolving the ionomer distribution in fuel cells using X-ray or electron microscopies is related to the low mass-thickness contrast with respect to the carbon substrates. Conventionally, this has been addressed using contrast-enhancing sample preparation steps such as staining[8] or ion-exchange[9–11], or analytical techniques sensitive to fluorine, e.g., energy dispersive spectroscopy (EDS)[2,12], electron energy loss spectroscopy (EELS)[2], energy-filtered (EF)-TEM[10,13], and

X-ray spectromicroscopy[12,14–17]. Analytical techniques are usually highly dose-intensive which can result in severe ionomer degradation from radiolysis. Therefore, ion-exchange was previously used in combination with electron tomography and high angle annular dark field scanning (HAADF-S)TEM to map ionomer coverage on carbons[11]. It nevertheless bears inherent limitations due to the intensity of the ion-exchanged ionomer phase, which prevents the analysis of samples with nanocatalysts. This complicates conclusions as ionomers interact preferentially with the Pt surface[18], potentially leading to discrepancies between model samples and catalyst layers. Moreover, the effect of the ion-exchange step on the ionomer (nano-)morphology remains uncertain, and discrepancies and swelling have been observed after ion-exchange[13]. We have also found that this protocol leads to a variety of results in catalyst layer samples, which we demonstrate in Supplementary Fig. 12 with two representative areas of a model, Pt-free, catalyst layer fabricated with Nafion D2021 and Vulcan carbon blacks at 0.7 w/w I/C, ion-exchanged with Cs^+ ions following a procedure discussed elsewhere[19], and imaged in HAADF-STEM. These areas exhibited two distinct morphologies which we term here “continuous” and “clustered”. In the clustered case, crystallites, 2-3 nm wide, are observed. High resolution images (not shown) indicate a lattice spacing corresponding to CsF and point towards a byproduct of e-beam induced degradation, release of fluorine radicals by radiolysis, and reaction with the metal cations. Alternatively, some areas exhibited the expected continuous morphology, with no crystallites observable but evidence of a relatively homogeneous and amorphous layer surrounding the carbons. We hypothesize these discrepancies arise from the presence of locally thicker ionomer layers and patches, as shown in the main manuscript, which would provide sufficient reactant for CsF to arrange in crystallites. Additionnally, a locally different ionomer nanomorphology could also have an influence, as ionomers are known to exhibit a transition between 10 and 20 nm from bulk-like behavior with phase separation to dispersion-like with weak phase separation, characteristic of ultra-thin layers[20,21].

It appears therefore that a representative investigation of the ionomer coverage using such a sample preparation procedure is complex. Indeed, the crystallites would challenge the analysis of samples that contain Pt nanoparticles due to their similar shape. Moreover, they do not appear in tomographic reconstructions as a continuous phase (Supplementary Fig. 12), potentially inhibiting the precise identification and segmentation of the ionomer phase. As a consequence, our work focused on using bright field TEM and phase contrast as a mean to image pristine catalyst layers without contrast-enhancing sample preparation steps that can lead to erroneous interpretation of the results.

Supplementary Note 5: Pt utilization in low RH conditions

The electrochemical oxidation of CO chemisorbed onto a Pt surface, *CO stripping*, is well-suited to reliably determine the electrochemically accessible surface area (ECSA) of Pt/C catalysts, because the adsorption strength of CO ensures near-complete coverage[22]. As CO cannot be electrooxidized in the absence of water and a proton-conducting pathway, the obtained ECSA is dependent on the availability of both within the Pt/C catalyst layer, which is typically controlled *via* the relative humidity.

Three processes are primarily responsible for the availability of liquid water in a Pt/C catalyst layer: Water uptake by the ionomer's sulfonate end groups, capillary condensation in the carbon particles' internal pores, and wetting the Pt surface via water vapor adsorption[23]. The ionomer's sulfonic acid sites are solvated by a hydration shell even at very low RH (<20 %), and thus a Pt surface in direct contact with the ionomer can electrochemically oxidize (strip) chemisorbed CO at such low humidification levels. For solid carbon supports, where Pt nanoparticles are predominantly present on the external surface of carbon particles and thus fully in contact with the ionomer phase, all Pt nanoparticles are active for CO stripping throughout the entire relative humidity range, i.e., the Pt utilization is always close to 100%.

In porous, high surface area carbon supports, such as the Ketjenblack carbon used in this study, a significant fraction of Pt nanoparticles is hosted within internal pores, which are not accessible to ionomer strands due to size exclusion. At high RH above 70%, capillary condensation in these pores ensures that all Pt particles are contacted by liquid water and capable of CO stripping, even though the ionomer is not present everywhere. This equates to a Pt utilization of 100%. Upon decreasing RH, the extent of capillary condensation is gradually reduced and ceases completely between 40-60% RH[24]. Below this RH, one would expect the ECSA, and accordingly the Pt utilization, to reach a plateau corresponding to the fraction of external Pt surface in direct contact with the ionomer. This, however, is virtually never observed in the existing literature[25–27]. In most cases, the Pt utilization starts to decrease at intermediate RH and then falls monotonically until it reaches the lower experimental cutoff value for the RH (10-25% RH), typically determined by the technical limits of the humidifying system. Chowdhury and co-workers explained this phenomenon through continuous water bridges between adsorbed thin films on Pt nanoparticles. If the interparticle distance of dispersed Pt, scaling with the Pt weight fraction, is sufficiently small, these bridges may supply water and protons to Pt surfaces that are not in direct contact with the ionomer. Crucially, this occurs at much lower RHs than would be required for capillary condensation of liquid water, because both ionomer water uptake and Pt wetting are non-zero even below 20% RH. A plateau in Pt utilization is therefore only observed when the bridging function is hindered by sufficiently large Pt interparticle distances (e.g., a 10 $wt\%_{Pt}$ catalyst[26]) or severely disrupted ionomer coverage (e.g., I/C ratio of 0.1[28]).

We thus suggest that Pt utilization measurements at low RH only approximate the true dry state corresponding to 0% RH, which is inaccessible to the CO stripping methodology because the oxidation reaction requires water. Above 0% RH, Pt surfaces not immediately contacted by the ionomer gradually become accessible *via* water bridges, even before the onset of liquid water condensation on the carbon surface itself. The Pt utilization therefore never reaches a true plateau equivalent to the connected surface found in cryogenic TEM tomography. Our obtained Pt utilization of 31% at 10% RH suggests that at these levels of humidification, the electrochemically accessible Pt surface is area is slightly larger than that found to be in direct ionomer contact in TEM, i.e., 15% at 0% RH, but below the value of the total surface of connected particles, yielding 52% Pt utilization, as detailed in Figure 3 of the main text.

Supplementary Figures

Supplementary Figure 1: dose-series

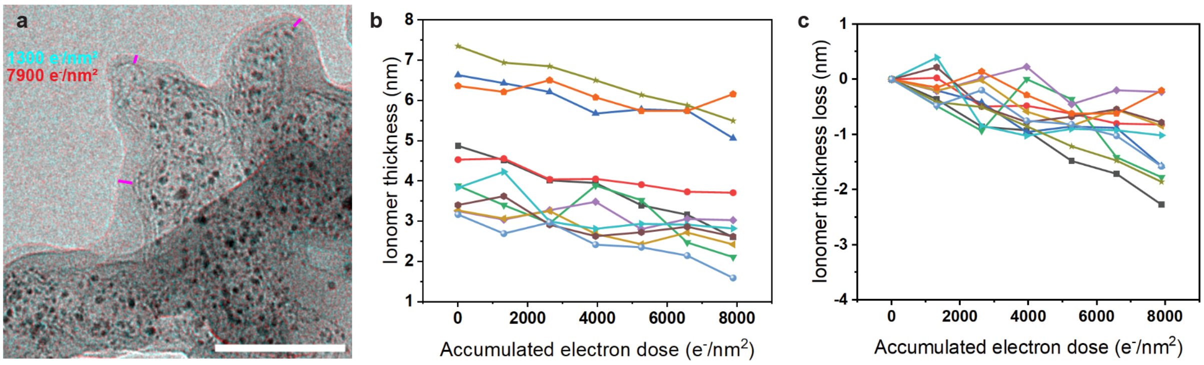

Supplementary Fig. 1 | Ionomer degradation analysis at cryo-temperature. **a** Composite BF-TEM image of a typical area used for measurements of the ionomer thickness as a function of accumulated electron dose. Some of the points of the measurements are highlighted by magenta makers. Cyan channel is the image at 1300 e^{-}/nm^2, Red channel is at 7900 e^{-}/nm^2. Scale bar is 50 nm. **b** Ionomer layer shrinkage for N = 11 measurements and **c** corresponding average thickness loss.

Supplementary Figure 2: e-beam induced damages during tilt-series acquisition

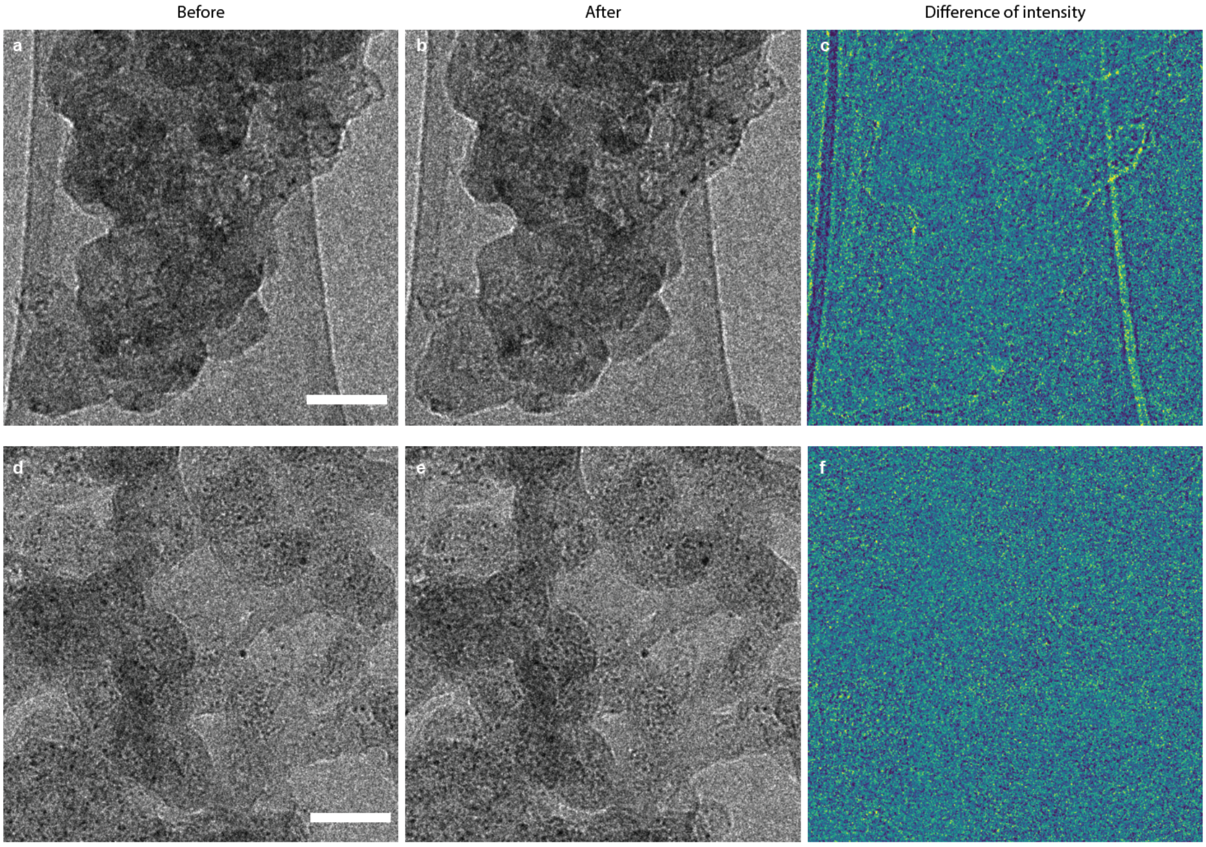

Supplementary Fig. 2 | Comparison of sample shrinkage from e-beam irradiation during tilt-series acquisition. a-c, Aggregate from a dispersion of the LSC7 sample imaged before **a** and after **b** tilt-series acquisition, totaling 3600 $e^{-}.nm^2$, and **c** difference of intensity between the two images after alignment. Increasing contrast indicates a greater shift during acquisition Similarly, **d-f** depicts the area of a microtomed section from the HSC7 sample before **d** and after **e** tilt-series acquisition, totaling 3450 $e^{-}.nm^2$, and **f** difference of intensity between the two images. Scale bars are 50 nm.

Supplementary Figure 3: workflow of cryo-CARE denoising

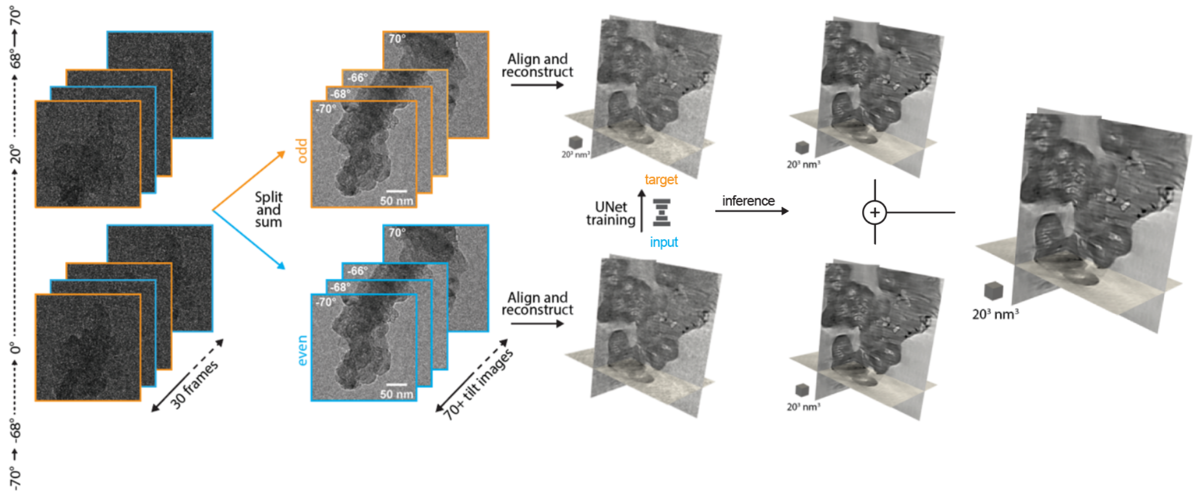

Supplementary Fig. 3 | Summarized workflow of cryo-CARE denoising showing data preparation, and DL model training and inference.

Supplementary Figure 4: morphology of graphitized Vulcans

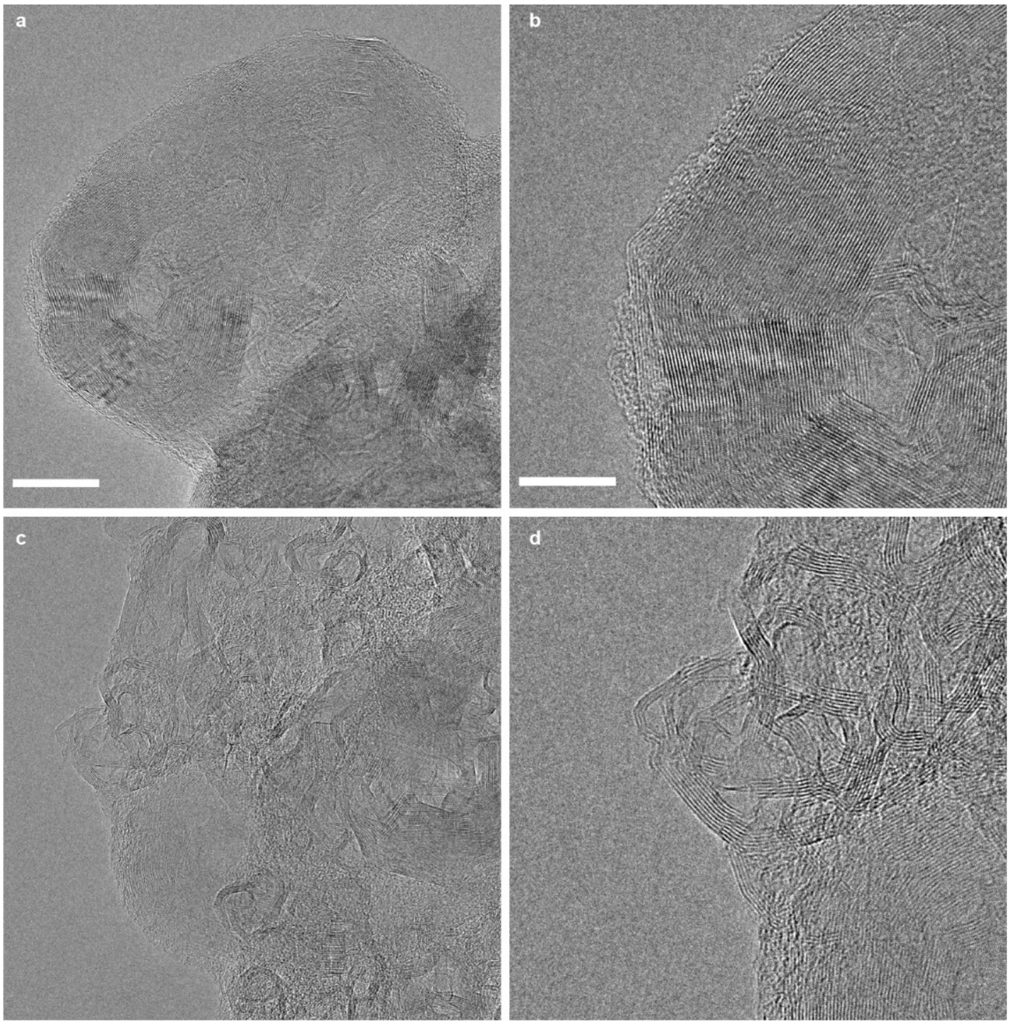

Supplementary Fig. 4 | graphitized Vulcan morphologies. a, c, HRTEM micrographs of aggregates and particles representative of the various morphologies in a graphitized Vulcan-type carbon black as used to fabricate samples LSC3, 7 and 12. Scale bar is 20 nm. **b, d**, Corresponding close-ups. Scale bar is 10 nm. The sample was prepared by dispersion from a model catalyst layer containing ionomer and imaged at high electron dose (> 10^7 $e^-.nm^{-2}$). Some highly degraded ionomer material is visible in **b** and **d**.

Supplementary Figure 5: EDS on HSC7 site

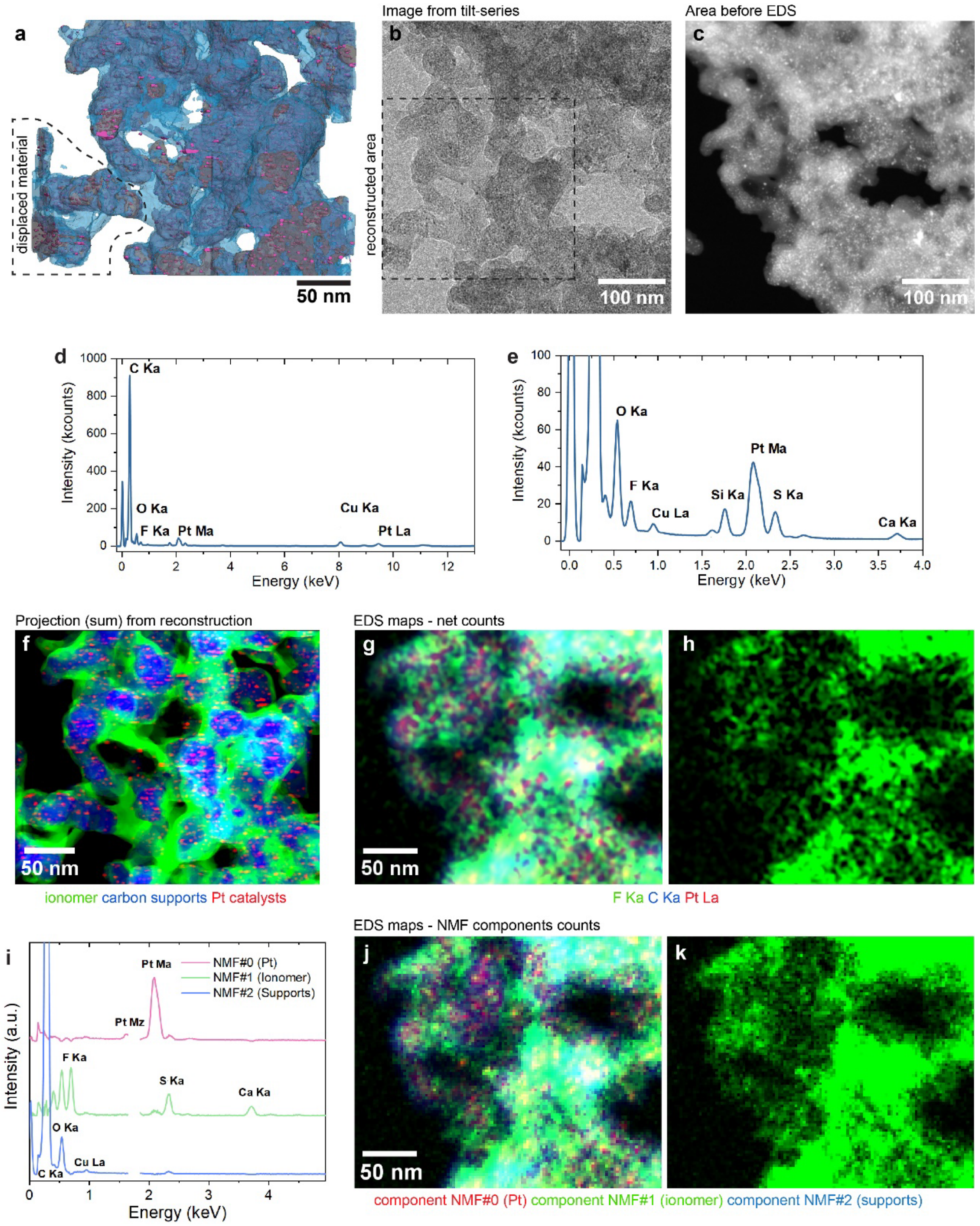

Supplementary Fig. 5 | EDS analysis of the site of the HSC7 reconstruction. a) 3D visualization of the reconstructed volume, oriented to match the geometry and orientation of the EDS acquisition. Comparison of the area as acquired b) in BF-TEM during the tilt-series and c) in STEM-HAADF before the EDS acquisition. e) EDS spectrum integrated over the whole area corresponding to the reconstruction and h) zoom-in on low-energy region. f) projection as the sum along the z-axis of the reconstructed volume. g) Composite EDS map with F Ka (green), C Ka (blue) and Pt L (red) net counts. h) isolated EDS map of the F Ka net counts. Acquisition at RT and 200 kV, with a 0.6 nA probe current, 100 µs dwell time and 0.6 nm pixels. The total dose was 4.9×10^7 e^-/nm^2. Data processed with a spatial gaussian pre-filter (σ = 3) and polynomial background removal. i-k) Results from decomposition with non-negative matrix factorization on data spatially binned by 4 in preprocessing. The Si Ka line was masked to avoid interference. i) components identified by the decomposition, with composition related to Pt catalysts (NMF#0), ionomer (NMF#1) and carbon supports (NMF#2). j) composite map of the spatial distribution of the identified components and k) isolated map of the component associated with ionomer.

Supplementary Figure 6: morphology of Ketjenblacks aggregates

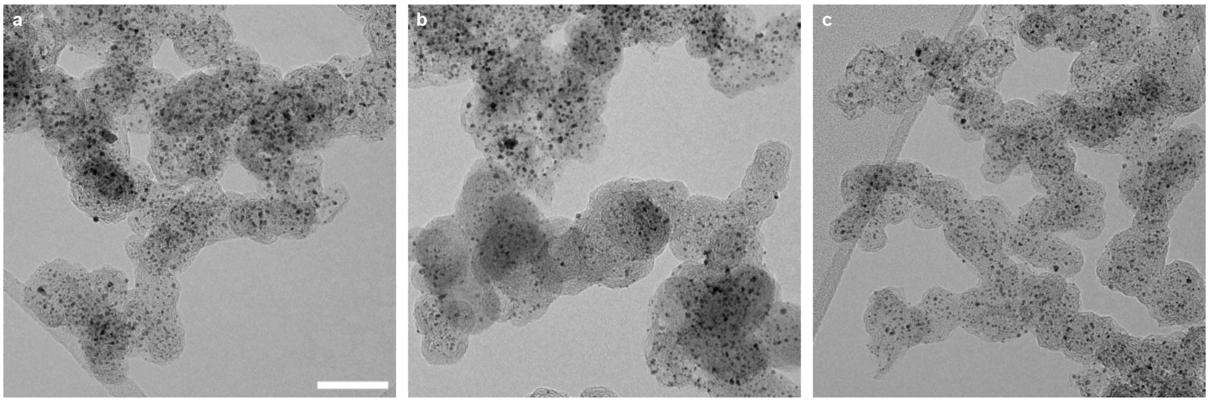

Supplementary Fig. 6 | Morphology of Ketjenblack catalysts. a-c, representative HRTEM of dispersed 19.8 $wt\%_{Pt}$ Ketjenblacks as used in sample HSC7. Scale bar is 50 nm.

Supplementary Figure 7: pores in Ketjenblacks

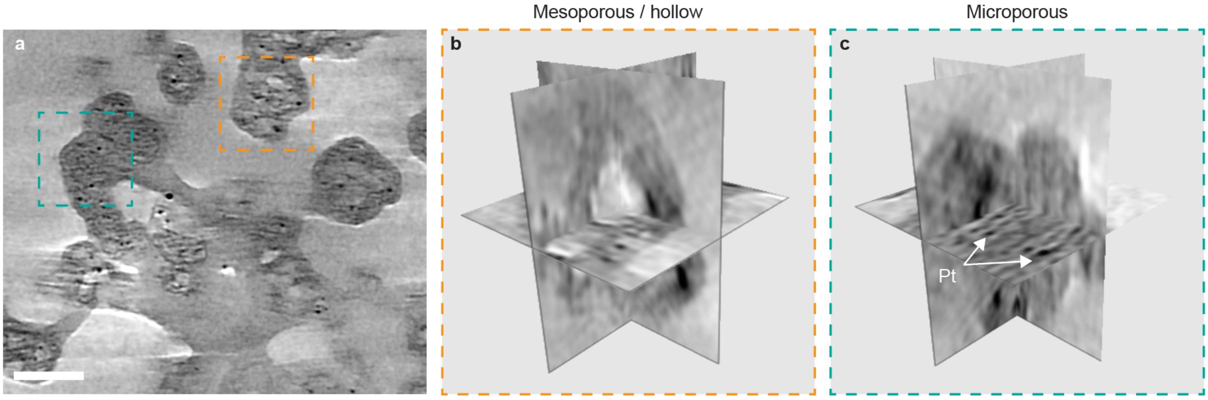

Supplementary Fig. 7 | Interior morphology of Ketjenblack catalysts. a, Tomogram from the cryo-ET reconstruction of the HSC7 sample presented in Fig. 2 of the main manuscript. Scale bar is 50 nm. Two categories of carbon particles can be found, with **b,** a mesoporous / hollow core directly visible and **c**, an interior microporosity demonstrated by the presence of Pt. Due to contrast and resolution limitations the porosity was not segmented.

Supplementary Figure 8: connected components analysis of the ionomer network

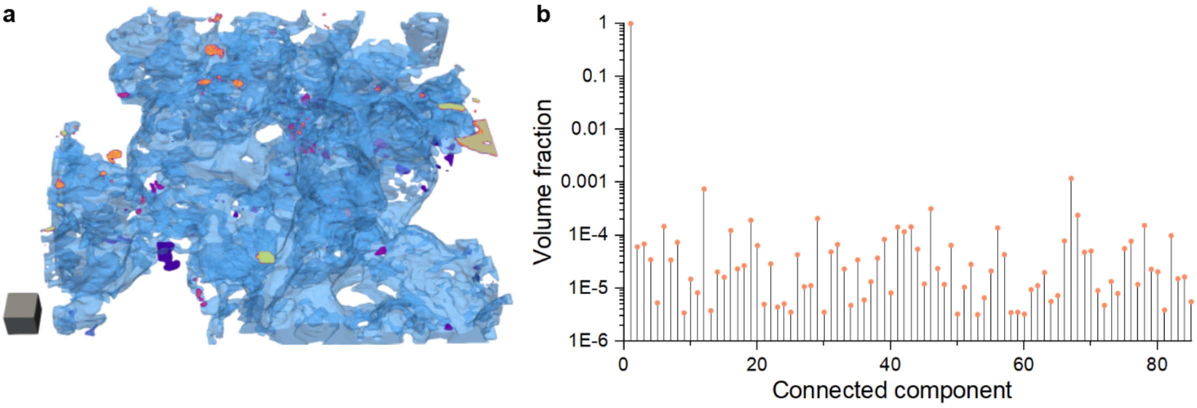

Supplementary Fig. 8 | Connected components analysis of the ionomer network. a, Labeled connected components in the ionomer phase of the cryo-ET reconstruction of the HSC7 sample presented in Fig. 2 of the main manuscript. Scale cube is 20^3 nm^3. **b**, Plot of the volume fraction for each identified component.

Supplementary Figure 9: ionomer morphologies in HSC7 catalyst layer

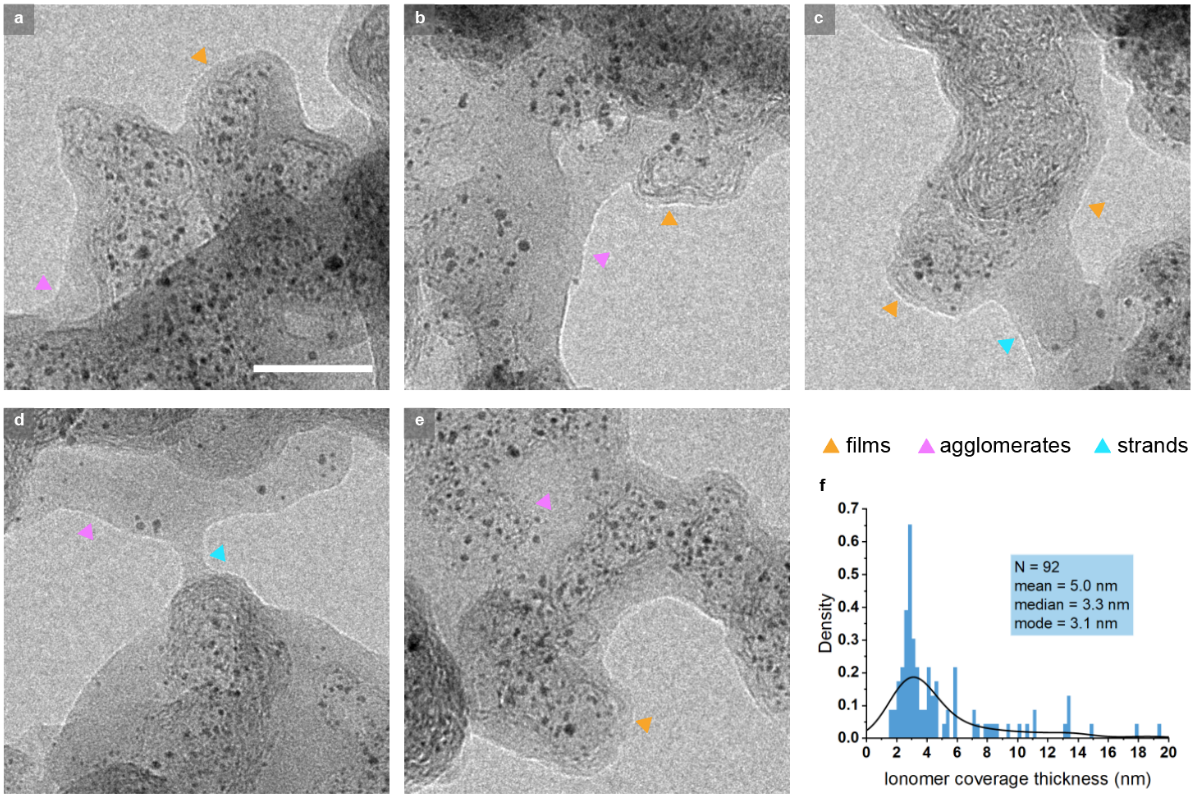

Supplementary Fig. 9 | Ionomer morphologies in catalyst layers. a-e, representative BF-TEM images of a microtomed section of the HSC7 sample, acquired in cryogenic conditions. The dose was 1200 – 1500 e^-/nm^2 per image. Morphologies similar to those observed in the tomographic reconstruction are identified with coloured markers. Scale bar is 40 nm. **f,** Histogram of the ionomer coverage thickness measured from the BF-TEM images. Black line is a kernel plot of the histogram

Supplementary Figure 10: representativeness of measurements within HSC7 volume

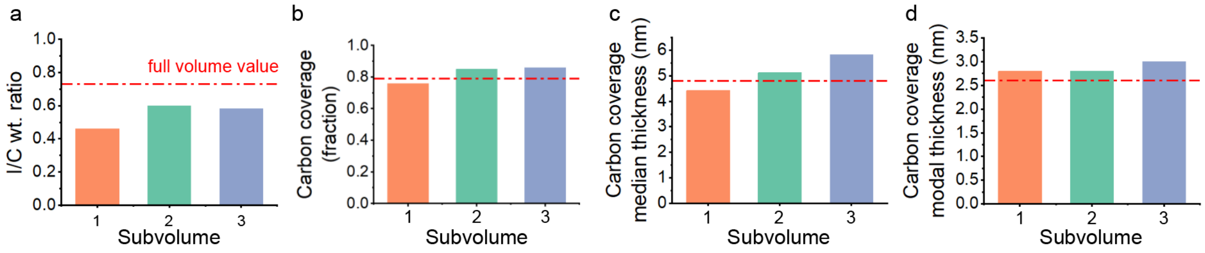

Supplementary Fig. 10 | Analysis of the representativeness of the measurements in the HSC7 reconstruction. Measurements of a) the I/C ratio, b) carbon surface coverage, c) median and d) modal thicknesses of the ionomer coverage on carbon surfaces. Measurements were performed on three sub-volumes of size 140 x 250 x 40 nm taken at the beginning, centre, and end of the volume (total size: 300 x 250 x 180 nm) used for Figure 2 of the main manuscript, along the z direction.

Supplementary Figure 11: electrode homogeneity

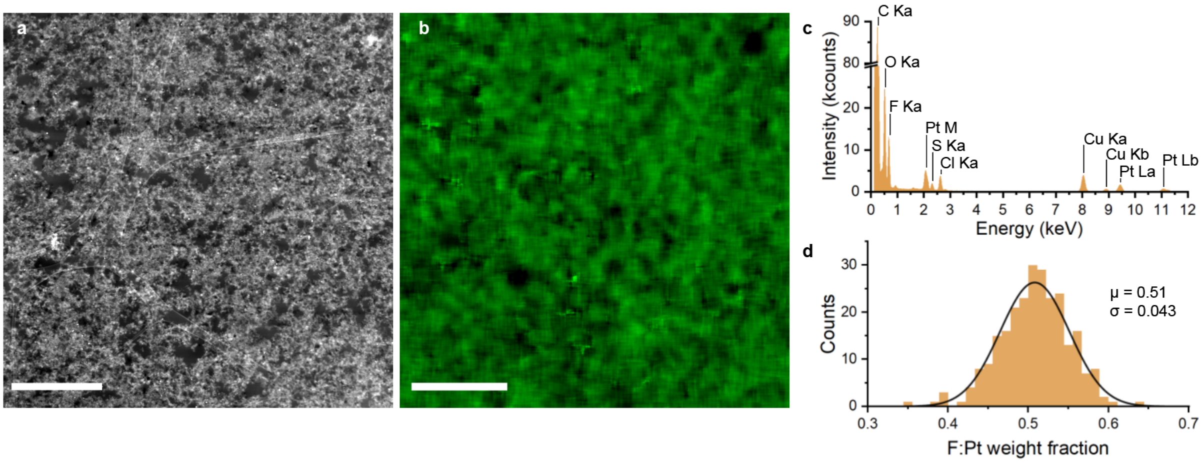

Supplementary Fig. 11 | Assessment of the ionomer distribution at the catalyst layer scale. a, HAADF-STEM image and **b,** corresponding F:Pt weight fraction map quantified from STEM-EDS. Acquisition was done on a microtomed cross-section of the HSC7 sample. Scale bars are 2 µm. **c,** Representative STEM-EDS spectrum. **d**, F:Pt weight fraction distribution calculated from 500^2 nm^2 tiles (N = 256) sampled from the map in **d**. Black line is a fitted normal distribution parameterized by its mean μ and standard deviation σ.

Supplementary Figure 12: ionomer morphology after ion-exchange

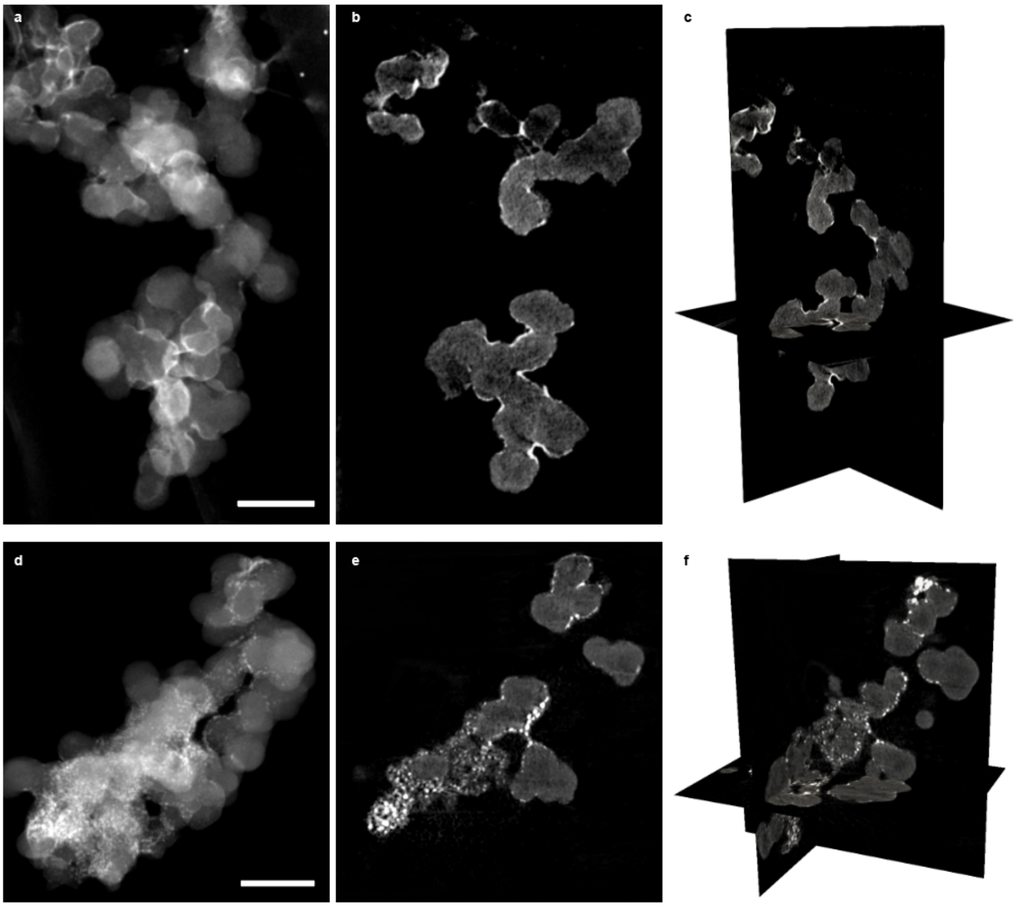

Supplementary Fig. 12 | Representative ionomer morphologies after ion-exchange. a, d, HAADF-STEM images from ionomer / carbon aggregates from a model, Pt-free catalyst layer fabricated with Vulcan XC72 carbon black and Nafion 2021 at 0.7 w/w I/C ratio and ion-exchanged with Cs^+ as discussed in Supplementary Text. Ionomer layers typically appeared "continuous" **a**, or "clustered" **d**. Scale bars are 100 nm. **b, e,** Corresponding tomograms showing a central section through the reconstructed volumes of the aggregates. **b**, Reconstruction performed by the weighted back projection (WBP) algorithm implemented with the ASTRA toolbox[29,30] and **e** with the model-based iterative reconstruction (MBIR) method[31]. **c, f**, Multi-orthoslice view of the reconstructions.

Supplementary Tables

Supplementary Table 1: composition of various catalyst layers

Sample denomination	Figure of reference	Carbon type	Pt loading (wt% / $mg.cm^{-2}$)	Ionomer type	Ionomer loading (I/C wr)
**LSC3**	Fig. 3a	Graphitized Vulcan (TKK)	8.7 / ~ 0.04	Nafion D2021	0.35
**LSC7**	Fig. 3b	Graphitized Vulcan (TKK)	8.7 / ~ 0.04	Nafion D2021	0.7
**LSC12**	Fig. 3c	Graphitized Vulcan (TKK)	8.7 / ~ 0.04	Nafion D2021	1.2
**HSC7**	Fig. 4	Ketjenblack (TKK)	19.8 / ~ 0.08	3M 800EW	0.7

Supplementary Table 2: tilt-series acquisition parameters

Sample denomination	Figure of reference	Sampling range (°) [acquired] / [used for reconstruction]	Dose rate ($e^-.nm^{-2}.s^{-1}$)	Total accumulated dose, incl. pre-irradiation ($e^-.nm^{-2}$)
**LSC3**	Fig. 3a	[-66, +74] / [-66, +72]	33	3600
**LSC7**	Fig. 3b	[-68, +76] / [-66, +76]	33	3750
**LSC12**	Fig. 3c	[-66, +74] / [-66, +66]	20	2230
**HSC7**	Fig. 4	[-62, +70] / [-62, +70]	33	3450

Supplementary References

1. Egerton, R. F. Radiation damage to organic and inorganic specimens in the TEM. *Micron* **119** 72–87 (2019)

2. Cullen, D. A. *et al.* Imaging and Microanalysis of Thin Ionomer Layers by Scanning Transmission Electron Microscopy. *J. Electrochem. Soc.* **161** F1111–F1117 (2014)

3. Legland, D., Arganda-Carreras, I. & Andrey, P. MorphoLibJ: integrated library and plugins for mathematical morphology with ImageJ. *Bioinformatics* **32** 3532–3534 (2016)

4. Domander, R., Felder, A. A. & Doube, M. BoneJ2 - refactoring established research software. *Wellcome Open Research* **6** 37 (2021)

5. Doube, M. Multithreaded two-pass connected components labelling and particle analysis in ImageJ. *Royal Society Open Science* **8** 201784

6. Gostick, J. T. *et al.* PoreSpy: A Python Toolkit for Quantitative Analysis of Porous Media Images. *Journal of Open Source Software* **4** 1296 (2019)

7. Cetinbas, F. C., Ahluwalia, R. K., Kariuki, N. N. & Myers, D. J. Agglomerates in Polymer Electrolyte Fuel Cell Electrodes: Part I. Structural Characterization. *J. Electrochem. Soc.* **165** F1051–F1058 (2018)

8. Xue, T., Trent, J. S. & Osseo-Asare, K. Characterization of nafion® membranes by transmission electron microscopy. *Journal of Membrane Science* **45** 261–271 (1989)

9. Rieberer, S. & Norian, K. H. Analytical electron microscopy of Nafion ion exchange membranes. *Ultramicroscopy* **41** 225–233 (1992)

10. Scheiba, F., Benker, N., Kunz, U., Roth, C. & Fuess, H. Electron microscopy techniques for the analysis of the polymer electrolyte distribution in proton exchange membrane fuel cells. *Journal of Power Sources* **177** 273–280 (2008)

11. Lopez-Haro, M. *et al.* Three-dimensional analysis of Nafion layers in fuel cell electrodes. *Nature Communications* **5** 5229 (2014)

12. Melo, L. G. A. *et al.* Quantitative Mapping of Ionomer in Catalyst Layers by Electron and X-ray Spectromicroscopy. *ECS Trans.* **80** 275–282 (2017)

13. Allen, F. I. *et al.* Morphology of Hydrated As-Cast Nafion Revealed through Cryo Electron Tomography. *ACS Macro Lett.* **4** 1–5 (2015)

14. Melo, L. G. A. & Hitchcock, A. P. Electron beam damage of perfluorosulfonic acid studied by soft X-ray spectromicroscopy. *Micron* **121** 8–20 (2019)

15. Wu, J. *et al.* 4D imaging of polymer electrolyte membrane fuel cell catalyst layers by soft X-ray spectro-tomography. *Journal of Power Sources* **381** 72–83 (2018)

16. Wu, J. *et al.* High-Resolution Imaging of Polymer Electrolyte Membrane Fuel Cell Cathode Layers by Soft X-ray Spectro-Ptychography. *J. Phys. Chem. C* **122** 11709–11719 (2018)

17. Takao, S. *et al.* Observation of Degradation of Pt and Carbon Support in Polymer Electrolyte Fuel Cell Using Combined Nano-X-ray Absorption Fine Structure and Transmission Electron Microscopy Techniques. *ACS Appl. Mater. Interfaces* 11 (2018)

18. Hatzell, K. B., Dixit, M. B., Berlinger, S. A. & Weber, A. Z. Understanding inks for porous-electrode formation. *J. Mater. Chem. A* **5** 20527–20533 (2017)

19. Komini Babu, S., Chung, H. T., Zelenay, P. & Litster, S. Resolving Electrode Morphology's Impact on Platinum Group Metal-Free Cathode Performance Using Nano-CT of 3D Hierarchical Pore and Ionomer Distribution. *ACS Appl. Mater. Interfaces* **8** 32764–32777 (2016)

20. Kusoglu, A. & Weber, A. Z. New Insights into Perfluorinated Sulfonic-Acid Ionomers. *Chem. Rev.* **117** 987–1104 (2017)

21. Modestino, M. A. *et al.* Self-Assembly and Transport Limitations in Confined Nafion Films. *Macromolecules* **46** 867–873 (2013)

22. Garrick, T. R., Moylan, T. E., Carpenter, M. K. & Kongkanand, A. Electrochemically Active Surface Area Measurement of Aged Pt Alloy Catalysts in PEM Fuel Cells by CO Stripping. *J. Electrochem. Soc.* **164** F55 (2016)

23. Chowdhury, A., Darling, R. M., Radke, C. J. & Weber, A. Z. Modeling Water Uptake and Pt Utilization in High Surface Area Carbon. *ECS Trans.* **92** 247 (2019)

24. Iden, H., Sato, K., Ohma, A. & Shinohara, K. Relationship among Microstructure, Ionomer Property and Proton Transport in Pseudo Catalyst Layers. *J. Electrochem. Soc.* **158** B987 (2011)

25. Shinozaki, K., Yamada, H. & Morimoto, Y. Relative Humidity Dependence of Pt Utilization in Polymer Electrolyte Fuel Cell Electrodes: Effects of Electrode Thickness, Ionomer-to-Carbon Ratio, Ionomer Equivalent Weight, and Carbon Support. *J. Electrochem. Soc.* **158** B467 (2011)

26. Padgett, E. *et al.* Connecting Fuel Cell Catalyst Nanostructure and Accessibility Using Quantitative Cryo-STEM Tomography. *J. Electrochem. Soc.* **165** F173–F180 (2018)

27. Ramaswamy, N., Gu, W., Ziegelbauer, J. M. & Kumaraguru, S. Carbon Support Microstructure Impact on High Current Density Transport Resistances in PEMFC Cathode. *J. Electrochem. Soc.* **167** 064515 (2020)

28. Soboleva, T. *et al.* On the Micro-, Meso-, and Macroporous Structures of Polymer Electrolyte Membrane Fuel Cell Catalyst Layers. *ACS Appl. Mater. Interfaces* **2** 375–384 (2010)

29. van Aarle, W. *et al.* The ASTRA Toolbox: A platform for advanced algorithm development in electron tomography. *Ultramicroscopy* **157** 35–47 (2015)

30. Palenstijn, W. J., Batenburg, K. J. & Sijbers, J. Performance improvements for iterative electron tomography reconstruction using graphics processing units (GPUs). *Journal of Structural Biology* **176** 250–253 (2011)

31. Venkatakrishnan, S. V. *et al.* A Model Based Iterative Reconstruction Algorithm For High Angle Annular Dark Field-Scanning Transmission Electron Microscope (HAADF-STEM) Tomography. *IEEE Transactions on Image Processing* **22** 4532–4544 (2013)
